# Hypoxia-Responsive Fluorescent Prodrugs: Self-Reporting Drug Delivery or Activation in Living Systems

**DOI:** 10.3390/bios16100540

**Published:** 2026-09-27

**Authors:** Xiaomu Hu, Xiaoyu Song, Yijie Qiu, Wei Du

**Affiliations:** 1 Department of Pharmacy, 900TH Hospital of Joint Logistic Support Force of PLA, Fuzhou 350025, China; huxiaomu2010@163.com (X.H.); 2240456007@fjtcm.edu.cn (X.S.); 2250456020@fjtcm.edu.cn (Y.Q.); 2 New Cornerstone Science Laboratory, College of Chemistry, Fuzhou University, Fuzhou 350108, China

**Keywords:** hypoxia, prodrug, drug activation, drug delivery, fluorescence imaging

## Abstract

Hypoxia is a hallmark of numerous pathological conditions, such as cancer, drug-induced liver injury, and rheumatoid arthritis. Given the contrast in oxygen levels between pathological and normal tissues, hypoxia-activated prodrugs have been developed by incorporating hypoxia-responsive groups to achieve targeted therapy while minimizing toxicity to normal cells. Recently, the integration of drug delivery or activation (including triggered release) with fluorescence imaging has emerged as a promising strategy in prodrug design, enabling self-reporting of drug processes and facilitating preclinical evaluation of prodrug performance. This review provides a comprehensive overview of hypoxia-responsive fluorescent prodrugs capable of self-reporting drug delivery or activation/release via fluorescence readouts. We summarize recent advancements in such prodrugs, focusing on design strategies, drug release or activation mechanisms, fluorescence signal modulation, and biomedical applications, especially in cancer treatment. Furthermore, we discuss current challenges and future perspectives for hypoxia-responsive fluorescent prodrugs, as well as potential directions for the development of the next generation of hypoxia-responsive prodrugs.

## 1. Introduction

Hypoxia is a relative biological condition in which oxygen availability is insufficient for the metabolic requirements of a particular tissue. Physiologically low oxygen occurs in several normal organs, whereas persistent, severe, or spatially dysregulated hypoxia is associated with tumors, ischemic diseases, inflammatory disorders, and drug-induced tissue injury [1,2,3]. Among these pathological settings, tumor hypoxia has attracted particular attention because of its profound effects on tumor progression, metastasis, and therapeutic resistance. Tumor hypoxia itself is highly heterogeneous, manifesting as acute (perfusion-limited), chronic (diffusion-limited), or cycling hypoxia, each with distinct biological consequences. The rapid proliferation of tumor cells, abnormal vascular architecture, and limited efficiency of oxygen diffusion collectively contribute to the formation of hypoxic regions within solid tumors [4]. Furthermore, this hypoxic state subsequently stabilizes and amplifies hypoxia-inducible factor-1α (HIF-1α), which in turn promotes the formation of abnormal and dysfunctional neo-vasculature that fails to adequately restore oxygenation while also driving therapeutic resistance, DNA repair downregulation, metastasis, and proliferation [5]. Ultimately, this interplay between aberrant vascular architecture and HIF-1α-driven adaptive responses establishes a vicious cycle that perpetuates and exacerbates intratumoral hypoxia.

The hypoxic tumor microenvironment disrupts normal antioxidant defenses and elevates oxidative stress [6]. Concurrently, the Warburg effect shifts cancer cell metabolism toward aerobic glycolysis, while exogenous drugs or electron-donating agents potentiate redox cycling [5,6]. These combined effects drive overexpression of specific reductases (e.g., nitroreductase [NTR] and azoreductase) in hypoxic cells. Accordingly, the activity of relevant reductases, combined with their interaction with local oxygen availability, represents a functional indicator of hypoxia and provides an important basis for selective imaging and treatment of hypoxic regions. Enzyme-responsive bioreductive activation strategy can therefore be exploited not only to identify hypoxia-associated pathological microenvironments but also to confine drug activation to specific cells or tissues, offering potential means to reduce off-target exposure, assess drug activation, and guide precision therapy [7,8,9].

In recent years, hypoxia-activated prodrugs (HAPs) have emerged as a promising strategy for targeted drug delivery and controlled release. HAPs are generally relatively inert under normoxic conditions but can be activated in hypoxic environments through enzymatic or chemical reduction [10]. This selective activation mechanism enables preferential release of cytotoxic agents in hypoxic tissues, thereby reducing damage to normal oxygenated tissues. Over the past few decades, various HAPs have been developed, including tirapazamine, PR-104, and evofosfamide (TH-302) [11,12,13]. These compounds typically contain bioreducible functional groups, such as nitroaromatic moieties, amine N-oxides, quinones, or azo groups, which can be reduced by intracellular reductases under hypoxic conditions [10,14].

Although HAPs have demonstrated promising antitumor activity in preclinical studies, their clinical translation remains challenging. One major limitation is the difficulty of monitoring drug location and activation in living systems. Conventional prodrugs release active drugs in hypoxic environments, but this activation process is typically “invisible”, making it difficult to determine whether drug release occurs at the desired time and location [15]. Without reliable methods to track drug location and activation in vivo, it remains challenging to optimize therapeutic regimens or predict treatment outcomes.

To address this issue, researchers have recently proposed a class of hypoxia-responsive fluorescent prodrugs that integrate therapeutic functions and imaging capabilities [7]. They are systems that can respond to hypoxic conditions while providing spatiotemporal information on either drug delivery or drug activation/release based on fluorescence imaging. It should be noted that the activation/release of these prodrugs is hypoxia-dependent. This process is not confined to genuine oxygen-dependent bioreductive activation/release but also encompasses hypoxia-associated enzyme-responsive mechanisms and those driven by treatment-induced or treatment-amplified hypoxia. Moreover, different prodrug activation systems respond to varying oxygen concentration thresholds: genuine O_2_-dependent bio-reduction requires sufficiently low pO_2_ to suppress oxygen back-reaction, whereas hypoxia-associated enzyme-responsive systems rely on HIF-1α-upregulated enzyme expression rather than direct O_2_ sensing. Depending on the system design, the fluorescence signal in these systems can report either drug delivery or drug activation/release. In one category, the fluorophore remains constantly emissive, serving to track the biodistribution of the prodrug. In the other, the fluorescence signal is positively correlated with prodrug activation or drug release process, enabling real-time visualization of drug processes in living cells or tissues. Both categories are commonly referred to as “self-reporting fluorescent prodrugs”, as they enable spatiotemporal reporting of either drug delivery or drug activation/release, depending on the specific system. Fluorescence imaging offers several advantages for monitoring drug activation in biological systems, including high sensitivity, excellent spatial resolution, and the ability to visualize dynamic processes in living cells [8].

The molecular design of hypoxia-responsive fluorescent prodrugs typically involves three key components: a therapeutic drug payload, a hypoxia-responsive triggering unit, and a fluorescent reporter group [16]. These components are integrated through carefully designed chemical linkers or nanoplatforms that keep the drug or fluorophore in an inactive state under normal conditions while the fluorophore is either quenched or constantly emissive depending on the intended reporting mode. Upon exposure to a hypoxic environment or in the presence of hypoxia-associated enzymes, the hypoxia-responsive triggering group undergoes reduction to release or activate drugs. Depending on the system design, the fluorescence signal may exhibit various response modes, including signal maintenance, signal enhancement, signal attenuation, or wavelength shifts. In the design of hypoxia-responsive prodrugs, multiple mechanisms for fluorescence signal regulation have been developed (Figure 1). Among them, photoinduced electron transfer (PeT) is one of the commonly used mechanisms. In this mechanism, electron transfer between the hypoxia-triggering group and the fluorophore quenches the fluorescence signal, whereas fluorescence is restored after reduction of the triggering group [17]. In addition, fluorescence resonance energy transfer (FRET) has been widely employed to monitor drug release through donor–acceptor interactions [18]. Other mechanisms, such as intramolecular charge transfer (ICT) and aggregation-induced emission (AIE), have further expanded the available strategies for fluorescence signal regulation in prodrug design [19,20].

Over the past decade, rapid advances in chemical biology, fluorescence imaging, and nanomedicine have significantly promoted the development of hypoxia-responsive fluorescent prodrugs [21]. To date, various systems have been reported for imaging-guided chemotherapy, photodynamic therapy (PDT), and combination therapy in hypoxic tumors [22,23]. In addition to small-molecule prodrugs, diverse nanostructured platforms, such as polymeric nanoparticles, liposomes, and semiconducting polymer nanoparticles, have been developed to improve the delivery efficiency and imaging performance of hypoxia-activated systems [24].

Despite substantial progress, hypoxia-responsive fluorescent prodrugs still face several challenges before their broad clinical application can be realized. Tumor hypoxia exhibits pronounced spatial and temporal heterogeneity, making it difficult to achieve uniform drug activation or release [25]. Moreover, fluorescence imaging in the visible-light region suffers from limited tissue penetration depth [26]. Meanwhile, the complex molecular architectures of multifunctional prodrug systems may also pose challenges in large-scale synthesis, pharmacokinetics, and clinical regulation.

In this review, we systematically summarize recent advances in hypoxia-responsive fluorescent prodrugs capable of self-reporting drug delivery or activation (including triggered release) in living systems. First, we summarize the molecular design strategies for constructing hypoxia-responsive fluorescent prodrugs, including the selection of drug payloads, triggering groups, linkers, and fluorophores. Next, we discuss hypoxia-triggered drug activation/release mechanisms and fluorescence signal-regulation strategies used to monitor these processes, followed by a summary and comparison of representative fluorescent prodrug systems. Finally, we discuss current challenges and future directions for the development of next-generation hypoxia-responsive fluorescent prodrugs.

## 2. Design Strategies for Hypoxia-Responsive Fluorescent Prodrugs

The development of hypoxia-responsive fluorescent prodrugs requires the integration of therapeutic and diagnostic functions within a single platform. Unlike conventional prodrugs that release active drugs only under specific conditions, hypoxia-responsive fluorescent prodrugs are designed to generate fluorescence signals while delivering therapeutic agents, thereby enabling real-time reporting of drug location or the drug activation/release process. Typically, a hypoxia-responsive fluorescent prodrug comprises four major components: (i) a therapeutic drug payload, (ii) a hypoxia-sensitive triggering unit, (iii) a linker that connects with the functional payload, such as a drug or fluorophore, and (iv) a fluorescent reporter capable of producing a detectable optical signal (Figure 1). These components are integrated through rational molecular design or supramolecular self-assembly so that the prodrug remains stable and inactive under normoxic conditions, whereas the fluorophore is either quenched or constantly emissive.

An important feature of hypoxia-responsive activation mechanisms is the competition between reductive activation and oxygen-mediated reoxidation. Under normoxic conditions, molecular oxygen can rapidly reoxidize reduced intermediates back to their original states, thereby preventing prodrug activation. In contrast, under hypoxic conditions, intracellular reduction can proceed continuously, resulting in the accumulation of activated intermediates [11,27,28]. This oxygen-concentration-dependent redox competition provides the molecular basis for the selective activation of hypoxia-responsive prodrugs or probes in low-oxygen tissues. The efficiency of this process is influenced by multiple factors, including the reduction potential of the triggering group, the activity of hypoxia-associated enzymes, and the local oxygen concentration. Therefore, an in-depth understanding of these activation mechanisms is essential for designing hypoxia-responsive fluorescent prodrugs with higher selectivity, improved activation efficiency, and enhanced imaging performance.

In this section, we summarize the key design elements for constructing hypoxia-responsive fluorescent prodrug systems and discuss how the roles of individual functional components and hypoxia-activation mechanisms collectively determine the overall performance of the prodrug systems.

### 2.1. Therapeutic Drug Payload

A therapeutic drug payload is the core component of a hypoxia-responsive fluorescent prodrug system, as it determines the pharmacological activity of the entire platform. Most reported HAPs incorporate highly cytotoxic chemotherapeutic agents. Although these drugs exhibit potent antitumor activity, their direct administration is often associated with severe systemic toxicity. Conversion into hypoxia-responsive prodrugs enables their selective activation in hypoxic tumor regions, thereby improving therapeutic selectivity and reducing adverse effects [29]. To date, a wide range of drugs have been exploited to construct hypoxia-responsive prodrugs (Figure 2).

Doxorubicin is one of the most used drug payloads in hypoxia-responsive prodrug systems, mainly owing to its potent anticancer activity and well-established mechanism of action. Doxorubicin exerts its cytotoxic effects primarily by intercalating into DNA and inhibiting topoisomerase II activity, which leads to DNA damage and induces apoptosis in rapidly proliferating cells [30]. In general, researchers mask the amino group of doxorubicin with hypoxia-sensitive moieties, thereby suppressing its cytotoxic activity in the prodrug state until activation occurs in hypoxic environments.

Camptothecin and its derivatives are also widely used as therapeutic agents in the design of hypoxia-responsive prodrugs. Camptothecin exerts its anticancer activity by inhibiting DNA topoisomerase I and inducing DNA single-strand breaks during DNA replication. Because camptothecin contains reactive functional groups that are amenable to chemical modification, it is particularly suitable for incorporation into prodrug systems through cleavable linkers [31].

In addition, commonly used therapeutic payloads include paclitaxel and nitrogen mustard derivatives. Paclitaxel disrupts microtubule dynamics and prevents cell division, whereas nitrogen mustard compounds induce interstrand DNA crosslinking through DNA alkylation [32]. Incorporation of these drugs into hypoxia-responsive prodrug systems enables their selective release in hypoxic regions of tumors or lesions.

### 2.2. Hypoxia-Sensitive Triggering Unit

Hypoxia-responsive triggers are key structural units that enable the selective activation of prodrugs in hypoxic environments. Under hypoxic conditions, hypoxia-triggered bioreductive reactions can induce the cleavage of specific chemical bonds, leading to the release of active drugs and potentially activating fluorescent signals [33,34]. Owing to the reduced oxygen concentration, intermediate reduction products are not rapidly reoxidized, allowing the reduction process to proceed efficiently.

Various chemical groups capable of undergoing bioreductive reactions under hypoxic conditions have been developed as triggering units, including nitroaromatic groups, azo groups, N-oxide groups, and quinone-based systems. Different types of trigger groups exhibit distinct reduction pathways and activation kinetics, thereby influencing the selectivity and efficiency of drug release [35].

#### 2.2.1. Nitroaromatic Groups

Nitroaromatic compounds are among the most widely used trigger groups in the design of hypoxia-responsive prodrugs, and their activation mainly depends on the catalytic activity of intracellular nitroreductases (NTRs). NTRs are flavin-dependent enzymes that typically use nicotinamide adenine dinucleotide (NADH) or nicotinamide adenine dinucleotide phosphate (NADPH) as electron donors. In hypoxic environments, these enzymes can catalyze the stepwise reduction of nitro groups, converting nitro groups (–NO_2_) sequentially into nitroso (–NO), hydroxylamine (–NHOH), and finally amino (–NH_2_) intermediates (Figure 3) [28,36,37]. This reductive transformation markedly alters the electronic properties of the molecule, converting the strongly electron-withdrawing nitro group into an electron-donating amino group, thereby inducing changes in molecular structural stability. Such changes often lead to linker cleavage or trigger self-immolative reactions, ultimately resulting in the release of active drugs or signaling molecules [38]. Therefore, nitroaromatic groups are of great value in the design of HAPs and molecular probes.

Among various nitroaromatic derivatives, nitroimidazole derivatives have attracted considerable attention and have been widely applied in the design of hypoxia imaging probes and HAPs because of their pronounced hypoxia selectivity. For example, Mark Matteucci and colleagues reported the design of hypoxia-activated achiral bioreductive phosphoramidate mustard prodrugs (e.g., **1**), in which phosphoramidate mustard cytotoxins with DNA-alkylating and cross-linking activity were conjugated to nitroheterocyclic triggering groups (specifically 2-nitroimidazole derivatives) that are reducible under hypoxic conditions. (Figure 4) [39]. In normal aerobic tissues, even if the trigger undergoes initial reduction, it can be rapidly reoxidized by oxygen; therefore, the prodrug remains relatively stable overall, reducing toxicity to normal tissues. In hypoxic regions of solid tumors, however, the low oxygen partial pressure allows the nitro group to undergo sustained reduction by intracellular reductases to nitroso, hydroxylamine, or amine forms. This process alters the electronic properties of the molecule and induces bond cleavage or self-immolative reactions, ultimately releasing the active phosphoramidate mustard.

Besides the nitroimidazole group, nitrobenzene-derived groups are another commonly used class of nitroaromatic groups. Franck Suzenet and co-workers developed two fluorescent probes functionalized with nitrobenzyl groups for NTR [37]. In this system, fluorescence is restored via the reduction of the nitro group, allowing for the real-time reporting of changes in NTR enzymatic activity (Figure 5). They also compared NTR expression in A2058 cells under normoxic, hypoxic, and DFO-induced conditions, and the results showed that hypoxia-mimetic treatment could increase the levels of the related enzymes.

#### 2.2.2. Azo Groups

Azo groups, as structurally tunable functional units, offer unique advantages in the design of hypoxia-responsive systems. Azo groups are commonly used as fluorescence-regulating elements or structural switches. Owing to their strong electron-withdrawing character and cis–trans isomerization capability, azo groups can effectively quench fluorescence signals through PeT or conjugation effects, allowing the system to maintain low background output in the inactive state. When the azo structure is disrupted, or its electronic effects are altered, this quenching effect is abolished, resulting in signal recovery [40].

The azo bond (–N=N–) is often cleaved to form amine products in the presence of azoreductase (AzoR) and NAD(P)H dehydrogenase. Under low-oxygen conditions, this reduction-driven covalent bond fragmentation process is greatly promoted because of the elevated reduction potential [5]. In contrast, this process is significantly suppressed under normoxic conditions. In functional molecular systems, reductive cleavage of the azo bond is usually accompanied by dissociation of the molecular scaffold or conformational changes, thereby triggering subsequent chemical or biological effects [41]. Therefore, this type of reaction exhibits favorable microenvironment-dependent responsiveness and provides an important foundation for the development of controllably activatable therapeutic and diagnostic systems [9,42].

In addition, azo groups exhibit good synthetic accessibility and structural diversity, and their electronic properties and response behaviors can be finely tuned by varying the type and position of substituents. This high degree of designability endows azo groups with broad application potential in the construction of smart responsive materials, molecular probes, and prodrug systems.

In recent years, responsive systems constructed based on azo bond reduction mechanisms have been widely applied in studies of hypoxia-related diseases, particularly showing favorable selective activation properties in the tumor microenvironment. Kenjiro Hanaoka and co-workers employed the azo group as a hypoxia-sensitive unit and proposed a strategy distinct from conventional FRET- or PeT-based mechanisms [43]. By directly incorporating the azo group into the conjugated system of the fluorescent core, the authors developed two azorhodamine probes, **2** and **3** (Figure 6a). The probes were in an initially “off” state because of ultrafast cis–trans isomerization of the azo bond. Under hypoxic conditions, intracellular reductases reduce and cleave the azo bond, restoring the original fluorophore structure and producing a pronounced fluorescence “turn-on” signal. More importantly, the two probes exhibited different response thresholds to oxygen concentration: **3** mainly responded to severe hypoxia (≤1% O_2_), whereas **2** could detect milder hypoxia (≤5% O_2_) (Figure 6b–d). These findings indicate that azo groups are not only suitable as “triggers” in hypoxia-responsive systems but can also enable graded responses to different levels of hypoxia through molecular structural modulation.

#### 2.2.3. N-Oxide Groups

N-oxide groups are functional units with pronounced electronic regulatory capability and are commonly used in molecular design to modulate compound polarity and electron distribution. Owing to the coordination interaction between nitrogen and oxygen atoms, these groups generally exhibit relatively high polarity and hydrophilicity. Meanwhile, N-oxide groups exhibit good structural compatibility and can be incorporated into diverse organic scaffolds without substantially disrupting the original structural framework. This feature provides considerable design flexibility for constructing responsive molecular systems and offers an important strategy for achieving precise functional regulation.

Furthermore, they reduce the electron-donating ability of the adjacent nitrogen center, thereby exerting important effects on the overall chemical properties of the molecule. In the construction of functional molecules, N-oxides are often employed as “electronic regulatory switches”. Their presence can alter the degree of conjugation and charge distribution within a molecule, thereby affecting the emission behavior of fluorophores or the activity state of drug molecules [44,45]. For example, in fluorescent probes, the introduction of an N-oxide moiety can attenuate electron-donor ability and suppress fluorescence emission; after conversion of this group, the electronic structure of the molecule is restored, resulting in enhanced signal output [46].

Bio-reduction in tertiary amine N-oxides is a representative hypoxia-activated reaction, exhibiting markedly enhanced reactivity under low-oxygen conditions. This process is typically mediated by cytochrome P450 reductase and related reductase systems, in which electron transfer occurs in the presence of NADPH, converting the N-oxide into the corresponding tertiary amine. From a functional perspective, the conversion of N-oxides to tertiary amines can trigger a series of subsequent molecular events, including bond cleavage, rearrangement, or self-immolative processes, thereby enabling the release of active compounds or structural reorganization. Such reduction-driven cascade reactions exhibit high selectivity under hypoxic conditions and have therefore been widely employed in the construction of controllably activated drug systems [2].

In practical applications, prodrugs based on the N-oxide reduction mechanism have demonstrated considerable potential in cancer therapy [47]. For example, AQ4N, also known as banoxantrone, can be reduced in hypoxic tumor regions to AQ4, a highly cytotoxic metabolite, thereby enabling selective killing of tumor cells (Figure 7a) [48,49]. This strategy provides an effective approach for improving therapeutic selectivity while reducing systemic toxicity. Justin Kim and co-workers proposed a new class of enamine N-oxides as hypoxia-responsive triggering groups for the construction of HAPs and imaging probes (Figure 7b) [48]. Compared with conventional nitroaromatic and quinone-based prodrugs, these structures are smaller and more modular. Under hypoxic conditions, they can be reduced by heme proteins, leading to the simultaneous release of active drugs and potentially electrophilic fragments. Using a staurosporine prodrug (**4**) as a proof of concept, the authors demonstrated that this system could be selectively activated under hypoxic conditions, exhibiting favorable hypoxia-responsive properties (Figure 7c). In addition, this triggering unit can also be used in the design of imaging molecules (e.g., probe **5**) for detecting hypoxic states (Figure 7d). The study emphasized that HAPs and hypoxia-activated fluorescent probes depend not only on low oxygen levels but also on the expression levels of relevant reductases; therefore, molecular tools that integrate therapeutic and imaging functions are better suited to guide precision therapy.

### 2.3. Linker Design

In a hypoxia-responsive fluorescent prodrug system, the linker serves as a critical structural unit connecting the trigger group with the functional payload, such as a drug or fluorophore. Its physicochemical properties have a decisive impact on the overall performance of the system. An ideal linker should remain sufficiently stable under physiological conditions to avoid nonspecific release, while enabling efficient and controllable cleavage in response to specific stimuli.

Structurally, commonly used linkers connect to the drug or fluorophore via ester, carbonate, carbamate, or ether bonds. These linkage types differ in hydrolytic stability, enzymatic sensitivity, and electronic effects, thereby endowing the system with distinct release kinetic profiles [29,50,51]. Particularly, self-immolative linkers are specialized structures that undergo rapid cleavage through intramolecular cascade reactions after activation of a triggering unit, and they have been widely applied in hypoxia-responsive systems. Their defining feature is that the linker remains stable in the presence of an intact trigger group. However, once the trigger group is transformed or removed, intramolecular electronic rearrangement is initiated, leading to an irreversible fragmentation process [52].

Typical self-immolative processes often involve electronically driven elimination reactions, such as 1,4- or 1,6-elimination mechanisms. Among them, p-aminobenzyl-based structures, such as p-aminobenzyl alcohol (PABA)-derived linkers, are among the most widely used examples. Upon reduction or cleavage of the upstream trigger group, the electronic environment at the benzylic position is altered, thereby driving rapid linker fragmentation and releasing the payload molecule [53].

For example, Xiao and co-workers reported a novel ratiometric fluorescent compound, **6**, for tumor hypoxia detection [54]. **6** employs a p-nitrobenzyl group, a commonly used recognition unit in HAPs, which is linked to the fluorophore RHF through a carbamate bond. In hypoxic environments, NTR selectively reduces the nitro group and induces structural release, resulting in a fluorescence emission shift from blue to green (Figure 8a). Experimental results demonstrated that **6** exhibited a pronounced ratiometric signal change upon excitation at 410 nm, with more than a four-fold enhancement in fluorescence intensity at 550 nm within 10 min (Figure 8b). In cellular experiments, **6** was successfully applied for hypoxia imaging in A549 tumor cells and exhibited low cytotoxicity (Figure 8c).

### 2.4. Fluorophore Engineering

A fluorophore is an essential component of hypoxia-responsive fluorescent prodrugs, as it provides optical signals for reporting the drug activation process. Several factors should be considered when selecting fluorophores, including fluorescence quantum yield, photostability, biocompatibility, and excitation/emission wavelengths. Fluorophores commonly used in prodrug systems include coumarin, fluorescein, rhodamine, BODIPY, and cyanine dyes [9]. Among them, fluorophores with an emission in the near-infrared (NIR) window (700–1700 nm) are particularly important for bioimaging because NIR light enables deeper tissue penetration and can significantly reduce autofluorescence background in biological systems.

In addition to conventional fluorophores, AIE luminogens (AIEgens) have also been applied in hypoxia-responsive prodrug systems in recent years. Unlike traditional fluorophores, which often undergo aggregation-caused quenching (ACQ) in the aggregated state, AIEgens exhibit enhanced fluorescence emission upon aggregation. Therefore, they are particularly suitable for imaging studies of drug release processes in biological environments [55,56].

### 2.5. Integration of Therapeutic and Imaging Functions

The design of hypoxia-responsive fluorescent prodrugs aims to integrate both therapeutic and imaging functions into a single molecular system or nanoplatform. Achieving this goal requires the fluorescence readout to be spatiotemporally correlated with drug delivery or activation/release event. In ideally designed systems, two distinct reporting modes can be realized: (i) the fluorescence signal is generated simultaneously with, or immediately after, drug release/activation, enabling real-time visualization of the therapeutic process in a turn-on manner; or (ii) the fluorophore remains constantly emissive throughout the delivery process, allowing real-time tracking of prodrug biodistribution and accumulation, independent of the activation event. Both types of systems possess theranostic functions, regardless of whether the fluorescence readout is activation-dependent or constantly on [57].

With continuous advances in synthetic chemistry and molecular engineering, it is promising to construct increasingly sophisticated hypoxia-responsive fluorescent prodrug systems with excellent performance. These systems have shown substantial improvements in sensitivity, selectivity, and imaging capability. These advances provide important tools for investigating hypoxia-related diseases and demonstrate great potential for applications in precision medicine.

## 3. Fluorescence Signal Modulation Mechanisms

Fluorescence signal modulation is an important reporting mode of hypoxia-responsive fluorescent prodrugs, as it enables real-time visualization of drug activation/release processes in biological systems. In a rationally designed system, the fluorescence response is tightly coupled with prodrug activation, thereby ensuring that the fluorescence signal accurately reports the temporal profile of drug activation/release. Various photophysical mechanisms have been employed to construct activatable fluorescent prodrugs with high sensitivity and selectivity [58,59]. Overall, fluorescence modulation strategies generally rely on changes in electronic structure, molecular conformation, or intermolecular interactions induced by activation of hypoxia-responsive trigger groups. These changes alter the photophysical properties of the fluorophore, resulting in fluorescence “turn-on”, “turn-off”, or ratiometric responses.

### 3.1. Photoinduced Electron Transfer (PeT)

PeT is one of the most used mechanisms for constructing activatable fluorescent probes and prodrugs. In a PeT-based system, fluorescence quenching occurs when an electron donor or acceptor interacts with the excited-state fluorophore through intramolecular electron transfer (Figure 9). This process reduces fluorescence emission intensity until the electron-transfer pathway is disrupted [60].

In hypoxia-responsive fluorescent probes, PeT quenching is typically mediated by hypoxia-sensitive trigger groups. For example, nitroaromatic groups can function as strong electron acceptors and efficiently quench fluorescence through PeT mechanism. When the nitro group is reduced under hypoxic conditions, its electron-withdrawing ability decreases, thereby suppressing the PeT process and restoring fluorescence emission [61]. PeT-based systems generally produce pronounced fluorescence “turn-on” signals and exhibit high signal-to-background ratios. These features enable sensitive detection of hypoxia-related diseases.

For example, Wu and co-workers reported a dual-controlled fluorescent probe, **7**, for tumor hypoxia imaging (Figure 10) [62]. Probe **7** was developed by incorporating a nitrophenol group and a methyltetrazine moiety in a BODIPY scaffold. Both methyltetrazine and nitrophenol are recognized as quenching moieties in **7**. Specifically, m-nitrophenol induces fluorescence quenching via the PeT mechanism. The fluorescence signal of **7** was regulated by both NTR and light irradiation. In hypoxic tumor microenvironments, overexpressed NTR can reduce the nitro group and induce fluorescence recovery; subsequent blue light irradiation further removes the tetrazine-mediated quenching effect, resulting in a stronger fluorescence output. Experimental results showed that **7** exhibited more than a 60-fold fluorescence enhancement under the combined action of NTR and light, with high sensitivity and selectivity. Cellular experiments demonstrated that this probe could effectively distinguish hypoxic cells from normoxic cells and enable controllable imaging of hypoxic tumor regions.

### 3.2. Fluorescence Resonance Energy Transfer (FRET)

FRET is a widely used mechanism to monitor molecular interactions and biochemical processes. FRET occurs when an excited-state donor fluorophore transfers energy non-radiatively to a nearby acceptor fluorophore through dipole–dipole coupling (Figure 11). The energy transfer efficiency is highly dependent on the distance between the donor and acceptor molecules, typically occurring within a range of approximately 1–10 nm [63].

In hypoxia-responsive fluorescent prodrugs, FRET systems are generally constructed by linking a donor fluorophore to an acceptor or quencher via a hypoxia-sensitive linker. Under normoxic conditions, efficient FRET suppresses donor fluorescence. Upon activation of the prodrug in a hypoxic environment, cleavage of the linker separates the donor from the acceptor, thereby disrupting the FRET process and restoring fluorescence emission [64]. FRET-based systems are particularly useful for ratiometric imaging because the emission signals from both the donor and acceptor can be monitored simultaneously. This capability improves the accuracy of quantitative analysis and reduces interference from environmental factors.

Liu et al. developed a hypoxia-responsive fluorescent prodrug (TPMO-Az@G/R@CD) based on FRET for real-time monitoring of drug release in hypoxic cells [65]. The nanoprobe was constructed by encapsulating glycine and rhodamine B (RhB) within tetraphenylethylene (TPE)-embedded periodic mesoporous organosilica (PMO), with the pores capped by β-cyclodextrin (β-CD) via an azobenzene linker (**8**) that serves as the hypoxia-cleavable gatekeeper (Figure 12a). Under normoxic conditions, the close proximity between the TPE donor and RhB acceptor enables efficient FRET, resulting in dominant red emission (“turn-on”) and effective suppression of premature cargo leakage. Upon exposure to hypoxic cells, elevated azoreductase activity will cleave the azo bond, triggering dissociation of the β-CD cap and concurrent release of glycine and RhB. The consequent donor–acceptor separation disrupted FRET, leading to increased green TPE fluorescence and decreased red RhB emission. In SH-SY5Y cells, the RhB/TPE intensity ratio decreased progressively over 4 h under hypoxia conditions, whereas it remained largely unchanged under normoxia, demonstrating selective glycine release in hypoxic cells (Figure 12b,c).

### 3.3. Intramolecular Charge Transfer (ICT)

ICT is another important signaling mechanism commonly employed in activatable fluorescent probes [66]. ICT-based fluorophores are typically composed of an electron-donating group and an electron-accepting group connected through a conjugated π-system. Upon molecular excitation, electrons are transferred from the donor to the acceptor, generating a charge-separated excited state that determines the fluorescence emission wavelength and intensity [2]. When the target analyte binds to one terminus of the electron-accepting moiety, the group’s electron-withdrawing and -donating capacity intensifies, leading to a bathochromic shift in both absorption and fluorescence spectra. Conversely, if the analyte interacts with one end of the electron-donating unit, this interaction attenuates the group’s electron-donating and -accepting efficacy (Figure 13) [67]. ICT-based probes are particularly suitable for ratiometric imaging, as the spectral shifts induced by ICT modulation can be detected by measuring fluorescence intensities at two distinct wavelengths. This strategy can improve detection accuracy in complex biological environments.

In hypoxia-responsive fluorescent prodrugs, activation of the trigger moiety can alter the electron-donating or electron-accepting properties of the molecule, thereby modulating the ICT process. These changes usually lead to pronounced variations in fluorescence emission wavelength or intensity [68]. For example, Zhou and co-workers developed a novel hypoxia-targeted NIR multifunctional fluorescent probe, **9**, for hypoxia monitoring and tumor-targeted therapy [69]. In the presence of azoreductase, **9** released a NIR fluorophore and an antitumor nitrogen mustard drug (Figure 14a). Upon release of the fluorophore, the ICT effect was activated, resulting in a significant enhancement of red fluorescence intensity at 620 nm. Taking advantage of this property, dynamic imaging and monitoring of hypoxia in cancer cells and tissues could be achieved (Figure 14b). Under hypoxic conditions, the released antitumor drug promoted tumor cell apoptosis and inhibited tumor growth in mice (Figure 14c). As a multifunctional fluorescent probe, **9** successfully visualized hypoxic regions within tumors while simultaneously enabling in situ tumor therapy, demonstrating its potential for future cancer treatment.

### 3.4. Aggregation-Induced Emission (AIE)

AIE is a relatively recent fluorescence mechanism that has attracted considerable attention in the field of biomedical imaging. In contrast to conventional fluorescent molecules, which often suffer from the ACQ effect in the aggregated state, AIEgens exhibit weak fluorescence in dilute solutions but emit strong fluorescence upon aggregation (Figure 15a,b) [70]. The fundamental mechanism of AIE is generally attributed to the restriction of intramolecular motion (RIM). In solution, free rotation of molecular substructures dissipates the excited-state energy through nonradiative pathways. By contrast, when molecules aggregate, intramolecular motion is restricted, thereby suppressing nonradiative decay and enhancing fluorescence emission.

In hypoxia-responsive fluorescent prodrug systems, AIE fluorophores can be rationally designed so that drug activation triggers molecular aggregation or structural transformation, leading to enhanced fluorescence emission [71]. For example, Han et al. reported supramolecular complexes, **TPA-H⊂SAC4A**, for dual-purpose hypoxia-activated bioimaging and PDT [72]. These supramolecular complexes comprise a sulfonated, hypoxia-responsive azocalix[4]arene (**10**) host and a cationic, AIE-active photosensitizer (TPA-H) (Figure 16a). Electrostatic binding between TPA-H and **10** effectively quenches both the fluorescence emission and ROS production of TPA-H. However, under low-oxygen conditions, the azo bonds in **10** undergo reduction to yield an aniline derivative, triggering the release of encapsulated TPA-H and restoring its pristine fluorescence and ROS-generating capacity (Figure 16a). Notably, once liberated, TPA-H exhibits dynamic intracellular redistribution (from the plasma membrane to mitochondria), enabling a real-time, self-monitoring PDT process (Figure 16b). In vivo tumor imaging and therapy results further demonstrate that this hypoxia-responsive supramolecular system achieves robust tumor suppression specifically under hypoxic microenvironments (Figure 16c,d). This approach is expected to circumvent the need for intricate molecular synthesis and offers a promising route toward effective image-guided PDT.

### 3.5. Fluorescence “Turn-On”, “Turn-Off”, and Ratiometric Strategies

Depending on molecular design, hypoxia-responsive fluorescent prodrugs can employ different fluorescence response modes. Fluorescence “turn-on” probes are among the most widely used systems because they generate strong fluorescence signals upon activation while maintaining extremely low background fluorescence under normoxic conditions. This feature enhances detection sensitivity and enables visualization of hypoxic regions in living systems. In contrast, fluorescence “turn-off” probes operate through the opposite mechanism, whereby the fluorescence signal decreases after activation. Although they are less commonly used, they can still provide valuable information in certain imaging studies. Ratiometric probes represent a more advanced strategy, in which two emission signals are generated simultaneously within the same system. By measuring the intensity ratio between the two signals, ratiometric imaging can minimize interference arising from probe concentration, excitation intensity, and environmental factors, thereby improving quantitative accuracy in bioimaging [73].

Zhang et al. reported an innovative hypoxia-responsive cyanine-based photocage capable of simultaneously generating dual-channel fluorescence upon light activation, enabling precise spatiotemporal control [74]. This oxygen-sensitive caging system was initially established using a retro-synthetic dialkylamine-substituted cyanine (**11**), which undergoes sequential retro-aldol cleavage and hydrolysis to yield a distinct photoproduct upon exposure to NIR light (> 650 nm) (Figure 17a). Building on this design, the therapeutic potential of such photocages was further explored through the rational engineering of a sequence-activated prodrug **12** (Figure 17b). This prodrug is sequentially activated by acidic pH and light irradiation under hypoxia, and produces dual-channel fluorescence with maximum emission peaks at 810 nm and 530 nm upon excitation at 720 nm and 480 nm, respectively (Figure 17b,c). Upon accumulation in acidic tumor microenvironments, **12** transitions into a protonated, dissociated form, exhibiting strong NIR fluorescence at 810 nm (Fluorescence Channel 1; Figure 17d). Subsequently, under hypoxic tumor conditions, NIR light irradiation induces photolytic uncaging, accompanied by a dramatic spectral shift from 810 nm to 530 nm (Fluorescence Channel 2; Figure 17e), thereby enabling real-time, background-free visualization of camptothecin (CPT) release. This hypoxia-dependent NIR photocage strategy enables orthogonal remote control with spatiotemporal and dose precision, facilitating site-specific manipulation for in vivo targeted therapy.

### 3.6. Design Considerations for Fluorescence Modulation

When designing hypoxia-responsive fluorescent prodrugs based on fluorescence signal modulation mechanisms, multiple factors should be comprehensively considered. First, the fluorescence response should be closely coupled with the drug activation/release process. Second, the selected fluorophore should exhibit high brightness, good photostability, and excellent biocompatibility. Third, the excitation and emission wavelengths are preferably located in the NIR region to enhance tissue penetration and reduce interference from autofluorescence in biological systems.

With continued advances in fluorophore engineering and molecular design strategies, researchers have been able to construct increasingly sophisticated hypoxia-responsive fluorescent prodrug systems with superior performance, showing significant improvements in both sensitivity and imaging capability [74]. These systems provide powerful tools for investigating hypoxia-related biological processes and hold great promise for image-guided therapy.

## 4. Advancements in Hypoxia-Responsive Fluorescent Prodrugs

Over the past decades, numerous hypoxia-responsive fluorescent prodrugs have been developed for image-guided therapy and real-time monitoring of drug delivery or drug activation/release processes in living systems [14,75,76]. These systems typically integrate a hypoxia-responsive trigger, a fluorescent reporter, and a therapeutic drug payload within a single molecular platform or nanoplatform. Depending on the molecular design, these systems can report drug delivery or activation through distinct fluorescence readouts.

Representative hypoxia-responsive fluorescent prodrug systems can generally be classified according to the type of hypoxia-responsive trigger employed. Among them, nitroaromatic-based systems have been the most extensively investigated because they can be efficiently reduced by NTRs and exhibit favorable hypoxia selectivity. Other commonly used trigger groups include azo bonds and N-oxide moieties. In addition, with the development of nanotechnology, hypoxia-responsive nanotheranostic platforms that combine drug delivery with fluorescence imaging capabilities have also emerged in recent years [77].

Importantly, the fluorescence signal in these systems does not always report the same event. In some molecular prodrugs, fluorophore recovery and payload release arise from the same cleavage reaction, and fluorescence can therefore serve as a relatively direct qualitative indicator of drug release or activation. In other systems, fluorescence mainly reports a related event, such as reductase activity, nanocarrier disassembly, photosensitizer activation, or changes in the local hypoxic microenvironment. In these cases, the fluorescence signal should be interpreted as a qualitative indicator rather than a quantitative measure of released drug. Table 1 summarizes representative systems and highlights these distinctions.

### 4.1. Nitroaromatic-Based Fluorescent Prodrugs

For these systems, the drug and fluorophore can be integrated into a single molecular platform. For example, Jong Seung Kim and co-workers developed a theranostic small-molecule-based prodrug **13** that can be programmatically activated under hypoxic conditions [78]. This prodrug comprises four functional modules: nitrobenzene serving as the hypoxia-responsive group, which is reduced by NTR to trigger bond cleavage; biotin as the tumor-targeting unit for recognizing cancer cells with high biotin receptor expression; the chemotherapeutic agent SN38 as the therapeutic unit; and a rhodol fluorophore as the imaging reporter (Figure 18a). Prodrug **13** can be selectively activated in hypoxic environments, leading to the synchronized SN38 release and fluorescence “turn-on” of the rhodol fluorophore (Figure 18a,b). Upon entering biotin receptor-positive HeLa cells, **13** remained relatively stable under normoxic conditions, but was selectively activated under hypoxia, thereby enabling hypoxia-controlled cytotoxicity and fluorescence imaging of tumor cells (Figure 18c,d). This hypoxia-responsive fluorescent prodrug system not only facilitates theranostics of tumor cells but also allows real-time fluorescence monitoring of drug release.

Besides drug and fluorophore-integrated systems, certain fluorescent prodrugs utilize therapeutic agents (e.g., photosensitizers) to self-report their activation under hypoxic conditions. For instance, the Xu group developed a hypoxia-activatable photosensitizer, **14**, by introducing a 4-nitrobenzyl group into a hemicyanine photosensitizer (Figure 19a) [79]. **14** exhibits quenched fluorescence and an inactive photodynamic effect. Upon entering hypoxic tumor cells, NTR reduces the nitro group of **14** and induces a self-immolative reaction that restores NIR and ^1^O_2_ generation capability, thereby enabling synchronous activation of imaging and therapy. Meanwhile, the molecule releases a highly reactive quinone methide intermediate, which can covalently bind to neighboring proteins, thereby reducing diffusion and metabolic clearance while significantly prolonging intratumoral retention (Figure 19b). Moreover, delivery via acid-responsive mPEG-b-PDPA nanomicelles enhanced tumor accumulation and cellular uptake. This strategy integrates low background signal, high selectivity, long-term imaging, and enhanced PDT efficacy, providing a new approach for precision diagnosis and treatment of hypoxic tumors.

### 4.2. Azo-Based Fluorescent Prodrugs

To date, several azo-linked fluorescent prodrugs have been reported for hypoxia imaging and therapy [85]. For example, Zhao et al. developed an azoreductase-responsive colon-targeted prodrug, **15** (Figure 20a) [80]. In this system, a podophyllotoxin derivative (4′-*O*-demethyl-4*β*-(4″-aminoanilino)-4-desoxy-podophyllotoxin, AdP) was linked to a NIR fluorophore (Cy) through an azo bond, allowing the prodrug to remain stable in the stomach and small intestine (Figure 20a). Upon reaching the colon, the azo bond was specifically reduced by azoreductase produced by gut microbiota, leading to the release of the active drug AdP and a NIR “turn-on” FL signal. In vitro results showed that the real-time monitoring of the AdP release process could be achieved via a fluorescence imaging method (Figure 20b,c). In vivo results demonstrated efficient colonic accumulation and drug release, thereby improving local therapeutic efficacy while reducing off-target exposure (Figure 20d).

In addition to the covalent strategy that conjugates fluorophores and therapeutic agents via an azo bond to construct hypoxia-activated theranostic prodrugs, noncovalent interactions have also been reported for the fabrication of azo-based fluorescent prodrugs. Shi and co-workers designed an azo-reduction-responsive prodrug, nitrogen mustard azocalix[4]arene (**16**), which was assembled with the NIR fluorescent dye CyNH_2_ through host–guest noncovalent interactions to form the theranostic prodrug, **NMAC4A-CyNH_2_** (Figure 21a) [81]. The host−guest interaction between **16** and CyNH_2_ leads to the NIR fluorescence quenching of CyNH_2_. Under hypoxic conditions, the azo bond in **16** undergoes reductive cleavage, which triggers the release of the active nitrogen mustard drug *N*,*N*-bis(2-chloroethyl)-1,4-benzenediamine (NM), while concurrently eliminating the fluorescence quenching of CyNH_2_ (Figure 21b). This enables the synchronized release of the drug and the “turn-on” of NIR fluorescence. Compared with conventional covalent theranostic prodrugs, this strategy offers simpler synthesis, more flexible assembly, and favorable stability, selectivity, and NIR fluorescence imaging capability, making it suitable for drug delivery in hypoxic tumors and real-time tracking of the release process.

Notably, certain small-molecule chemical drugs inherently exhibit intrinsic fluorescence. This fluorescence can be quenched by azo bonds, enabling the self-reporting of its release from azo-based fluorescent prodrugs under hypoxic conditions. Zhou et al. reported an SN-38-derived azo hypoxia-responsive theranostic prodrug, **17**, to quantify the extent of drug release in the hypoxic tumor region specifically with therapeutic intervention [82]. The prodrug **17** comprises an antineoplastic irinotecan analog SN-38 and a hypoxia-sensitive aryl-azo benzyl alcohol moiety (Figure 22A). Under reductive hypoxic conditions or in the presence of rat liver microsomes, the azo bond was cleaved to release the active drug SN-38, accompanied by an increase in fluorescence emission at 560 nm, enabling real-time monitoring of drug release (Figure 22A,B). Cellular studies showed that both the fluorescence signal and cytotoxicity of **17** were significantly enhanced under hypoxic conditions but remained weak under normoxic conditions, indicating good selectivity (Figure 22C,D). Furthermore, **17**’s ability to inhibit tumor growth was confirmed in a xenograft mouse model bearing 4T1 tumors (Figure 22E).

### 4.3. N-Oxide-Based Fluorescent Prodrugs

Owing to their selective reduction to tertiary amines, N-oxide groups have been widely employed in the design of hypoxia-responsive fluorescent probes [28]. Meng et al. reported an N-oxide-based molecular probe **18**, which can be converted to SN, thereby triggering both second near-infrared (NIR-II, 1000–1700 nm) fluorescence and photothermal effects (Figure 23a) [83]. This probe and thermosensitive H_2_S donor GYY4137 morpholine salt were encapsulated in bis-serotonin (bis-5HT) surface-modified liposomes, forming the multifunctional nanoplatform **5SGNPs** for the theranostic treatment of pulmonary embolism (PE). **5SGNPs** can react with myeloperoxidase (MPO) to form large aggregates, enabling selective targeting of the thrombotic site. In vitro and cellular experiments confirmed that hypoxia triggers activatable long-wavelength NIR-II fluorescence imaging for high-precision disease monitoring, as well as activatable PTT coupled with controlled H_2_S release (Figure 23a-c). Animal experiments showed that this nanoagent not only achieves highly sensitive and selective visualization of thrombosis depending on the combined PTT and H_2_S therapy but also delivers enhanced therapeutic efficacy for PE compared to the antithrombotic drug (Figure 23d).

### 4.4. Nanostructured Hypoxia-Responsive Fluorescent Prodrugs

In addition to small-molecule prodrugs, advances in nanotechnology have promoted the development of hypoxia-responsive fluorescent prodrug systems. A typical approach for constructing nanostructured hypoxia-responsive fluorescent prodrugs is self-assembly of hydrophobic hypoxia-responsive therapeutic agents and imaging probes with amphiphilic polymers [85]. For instance, Ding et al. reported a hypoxia-responsive supramolecular nanoprodrug featuring a cross-linked core [86]. This nanoprodrug was assembled through multiple noncovalent interactions, including π–π stacking, host–guest inclusion, hydrogen bonding, and hydrophobic forces, between the hypoxia-responsive dendritic drug–drug conjugates and β-cyclodextrin-grafted PEG-blockpoly(glutamic acid) block copolymer. Upon NIR light irradiation, tumor cells treated with the nanoprodrug underwent PDT and PTT, leading to intensified intratumoral hypoxia. The accelerated hypoxic microenvironment prompted disassembly of the supramolecular nanoprodrug and activation of the chemotherapeutic agent, thereby achieving synergetic anticancer efficacy.

Notably, owing to the unique properties of nanostructures, hypoxia-responsive therapeutics and imaging agents can exist as separate components without the need for covalent linkage. Kang et al. conjugated paclitaxel (PTX) with 4-nitrobenzyl chloroformate to yield a hypoxia-responsive prodrug, **19** (Figure 24a) [84]. Under hypoxic conditions, the nitrobenzyl moiety undergoes bioreduction, releasing active PTX and enabling hypoxia-controlled tumor chemotherapy. Subsequent co-assembly of **19** with an AIE luminogen (AIEgen, MTPETT) and Pluronic F-127 produced a theranostic nanoplatform (**MPNPs**), which was further camouflaged with macrophage cell membrane to afford a tumor-targeting theranostic nanoplatform, **M1-MPNPs** (Figure 24a). The AIEgen exhibits bright NIR fluorescence, photoacoustic (PA) signal, and PDT property. **M1-MPNPs** enables dual-mode fluorescence and photoacoustic tumor imaging, as well as combination immunotherapy driven by the potent PDT effect and self-accelerated chemotherapy. Upon light irradiation, the photosensitizer MTPETT generates reactive ROS and consumes oxygen, thereby exacerbating local tumor hypoxia and promoting **19** release. Furthermore, the combination of PDT and PTX chemotherapy induces immunogenic cancer cell death. Cellular, in vivo, and ex vivo results collectively demonstrated the excellent tumor-targeted fluorescence imaging capability of **M1-MPNPs** (Figure 24b-d). Moreover, **M1-MPNPs** effectively triggered robust antitumor immune responses, leading to suppression of the primary tumor as well as significant inhibition of metastatic tumor growth in female mice bearing 4T1 tumors (Figure 24e). The integration of hypoxia-responsive activation mechanisms with nanotechnology provides a highly promising direction for the development of next-generation theranostic systems.

In addition to the noncovalent encapsulation of therapeutic agents and fluorophores by amphiphilic polymers, covalent conjugation of these functional molecules to polymers represents another promising strategy for constructing nanostructured hypoxia-responsive fluorescent prodrugs. Specifically, certain semiconducting polymers can function simultaneously as therapeutic agents and fluorescence imaging agents. Accordingly, by incorporating hypoxia-responsive linkers into these semiconducting polymers, nanostructured hypoxia-responsive fluorescent prodrugs can be synthesized. For example, Cui et al. developed a semiconducting polymer-based nano-prodrug (**SPNpd**) for hypoxia-responsive synergistic anticancer treatment (Figure 25) [24]. This nano-prodrug is constructed via self-assembly of **20**, a brush-type amphiphilic semiconducting polymer that is covalently tethered to the chemotherapeutic agent IPM-Br via nitroreductase-cleavable linkers. Under 808 nm irradiation, **SPNpd** produces ^1^O_2_ for PDT. Concurrently, PDT-mediated oxygen consumption further exacerbates tumor hypoxia, which accelerates nitroreductase-induced cleavage of the linkers, subsequently promoting release of IPM-Br and inducing tumor cell death.

## 5. Biomedical Applications

Hypoxia-responsive fluorescent prodrugs have emerged as powerful tools for integrating molecular imaging with targeted therapy. By exploiting the hypoxic microenvironment commonly present in various pathological tissues, these systems enable selective drug activation and real-time monitoring of therapeutic processes. These features offer significant advantages for disease diagnosis, treatment guidance, and therapeutic evaluation. Recent studies have demonstrated that hypoxia-responsive fluorescent prodrugs can be applied in a wide range of biomedical scenarios, including tumor imaging, image-guided chemotherapy, combination therapy, and monitoring of drug activation in living systems [87,88].

### 5.1. Tumor Hypoxia Imaging

Hypoxia is a hallmark of many solid tumors and is closely associated with tumor progression, metastasis, and therapeutic resistance. Therefore, accurate visualization of hypoxic regions within tumors is essential for understanding tumor biology and optimizing therapeutic strategies. Hypoxia-responsive fluorescent prodrugs provide a convenient and noninvasive approach to imaging tumor hypoxia in living systems [89]. Most hypoxia-responsive imaging systems rely on the enzymatic reduction of nitroaromatic or N-oxide groups under low-oxygen conditions. These reactions trigger fluorescence activation, thereby enabling selective visualization of hypoxic tissues. For example, various nitroreductase-responsive fluorescent probes have been developed for the highly sensitive detection of hypoxic tumor cells. These probes remain nonfluorescent under normoxic conditions but generate intense fluorescence signals after enzymatic reduction in hypoxic environments [78,79,90]. NIR fluorescent probes are particularly attractive for tumor hypoxia imaging because NIR light offers deeper tissue penetration and lower background autofluorescence. Accordingly, many recently developed hypoxia-responsive fluorescent prodrugs incorporate NIR fluorophores to improve in vivo imaging performance [91,92].

### 5.2. Real-Time Monitoring of Drug Activation

The ability to monitor drug activation in real time is one important advantage of most hypoxia-responsive fluorescent prodrugs. Conventional chemotherapy generally lacks direct methods for visualizing when and where drugs are activated in vivo. In contrast, some fluorescent prodrug systems can generate optical signals that qualitatively indicate the activation process of therapeutic agents [93]. In most of these systems, the drug is conjugated to a fluorophore through a hypoxia-responsive linker. Upon exposure to hypoxic conditions, the linker is cleaved, releasing the active drug and restoring fluorescence emission.

These systems have been used to track drug activation in living cells, tumor tissues, and animal models. For example, fluorescent prodrugs based on doxorubicin or camptothecin have been developed for selective activation in hypoxic tumor regions [94,95]. By correlating fluorescence signals with therapeutic outcomes, researchers can gain insights into drug delivery efficiency, activation kinetics, and treatment responses [48,69,78,86]. Real-time monitoring of drug activation may also help identify mechanisms of drug resistance. For example, insufficient activation of hypoxia-responsive prodrugs may indicate inadequate enzyme expression levels or elevated oxygen concentrations in tumor tissues. This information not only enhances therapeutic precision but also can promote the development of individualized treatment strategies.

### 5.3. Combination Therapy

Hypoxia-responsive fluorescent prodrugs can integrate multiple therapeutic modalities to enable effective combination therapy. For instance, PDT and PTT have been combined with hypoxia-activated chemotherapy to enhance therapeutic efficacy [96]. In these systems, hypoxia-responsive trigger groups are incorporated into multifunctional nanoplatforms that simultaneously contain chemotherapeutic agents and photosensitizers. After accumulation in tumor tissues, these systems can generate ROS upon light irradiation while releasing cytotoxic drugs in hypoxic regions. This synergistic strategy can overcome some limitations of monotherapy and improve the overall therapeutic outcome [85]. Another promising strategy involves combining HAPs with immunotherapy. Because tumor hypoxia affects immune cell infiltration and immune checkpoint signaling pathways, targeting hypoxic regions may help enhance the efficacy of immunotherapy.

### 5.4. Applications Beyond Cancer

Although most current studies on hypoxia-responsive fluorescent prodrugs focus on cancer therapy, hypoxia is also involved in many other pathological processes, including ischemic diseases, inflammatory disorders, and bacterial infections [97]. Therefore, hypoxia-responsive imaging and therapeutic systems may have broader biomedical applications. For example, hypoxia-sensitive probes have been used for the theranostics of pulmonary embolism [84]. In addition, hypoxia-responsive antibacterial systems have been explored for targeting bacterial infections in oxygen-limited environments. With continued advances in molecular design and imaging technologies, hypoxia-responsive fluorescent prodrugs are expected to play an increasingly important role in precision medicine.

## 6. Challenges and Future Perspectives

Although considerable advances have been achieved in the development of hypoxia-responsive fluorescent prodrug systems, multiple critical challenges persist prior to the clinical translation of these platforms. Addressing these limitations will require continued advances in molecular design, imaging technologies, and biological evaluation. Future research is expected to focus on improving activation specificity, optimizing fluorescence performance, establishing reliable quantitative correlation between fluorescence signals and drug activation, enhancing therapeutic efficacy, and expanding biomedical applications [98].

### 6.1. Improving Hypoxia Selectivity and Activation Efficiency

A major challenge in the development of hypoxia-responsive fluorescent prodrugs is achieving high selectivity toward hypoxic cells and tissues. Although many bioreductive trigger groups preferentially respond to low-oxygen environments, partial activation may still occur under normoxic conditions. Such nonspecific activation can reduce imaging accuracy and compromise therapeutic selectivity [34,99,100]. Future studies should focus on designing trigger groups with optimized redox potentials and higher enzyme specificity. Rational molecular engineering and computational simulations may facilitate the identification of new trigger structures with higher activation thresholds and greater sensitivity to hypoxic conditions. In addition, integrating multiple hypoxia-responsive mechanisms into a single system may further improve selectivity.

### 6.2. Enhancing Optical Properties for In Vivo Imaging

Another important challenge is improving the optical performance of fluorescent prodrugs for deep-tissue imaging. Many conventional fluorophores emit in the visible region, where imaging is limited by poor tissue penetration and strong background autofluorescence. To overcome these limitations, the development of probes operating in the NIR-II (1000–1700 nm) window has become an important research direction [101,102,103]. Fluorophores emitting in the NIR-II region can substantially increase imaging depth and improve the signal-to-noise ratio. Incorporating such fluorophores into hypoxia-responsive prodrug systems may greatly enhance their in vivo imaging performance. In addition, improving photostability, brightness, and biocompatibility remains essential for long-term biological imaging [104].

### 6.3. Towards Quantitative Correlation Between Fluorescence Signals and Drug Exposure

Even with improved optical properties, establishing a reliable quantitative relationship between fluorescence signal and active drug exposure or delivery efficiency remains a major challenge. Future studies should determine the temporal and spatial correlation between fluorescence activation, linker cleavage, active drug release, and pharmacological response under physiologically relevant conditions. Standardized validation should include quantitative drug-release analysis, assessment of fluorophore and drug biodistribution/clearance, and calibration of fluorescence signals while accounting for pH, aggregation, protein binding, photobleaching, and tissue optical attenuation. The development of ratiometric, lifetime-based, or photoacoustic imaging strategies, together with multimodal imaging and pharmacokinetic modeling, may reduce signal variability and improve quantitative reliability. Such efforts are essential before fluorescence can be used as a robust surrogate for intratumoral active drug concentration and treatment response.

### 6.4. Integration with Nanotechnology

Nanotechnology provides new opportunities to improve the delivery efficiency and multifunctionality of hypoxia-responsive fluorescent prodrugs. Nanocarriers, such as polymeric nanoparticles, liposomes, and inorganic nanomaterials, can enhance drug stability, prolong circulation time, and increase tumor accumulation through the enhanced permeability and retention (EPR) effect [105,106]. Moreover, nanoplatforms can integrate multiple imaging modalities and therapeutic strategies into a single system. For example, nanoparticles can combine fluorescence imaging with photoacoustic imaging, magnetic resonance imaging, or positron emission computed tomography [107]. Such multimodal imaging systems can provide complementary information and improve diagnostic accuracy.

### 6.5. Toward Clinical Translation

Although many hypoxia-responsive fluorescent prodrugs have shown promising performance in cellular and animal studies, their clinical translation remains limited. This limitation is attributable to several factors, including complex synthetic procedures, potential toxicity, and insufficient pharmacokinetic data. Before these systems can be applied in clinical settings, their biosafety, metabolic profiles, and long-term toxicity must be comprehensively evaluated [108].

Another important issue is the heterogeneity of tumor hypoxia. Oxygen levels within tumors can vary substantially depending on tumor type, tumor size, and microenvironmental conditions. Therefore, future studies should explore personalized therapeutic strategies to optimize hypoxia-responsive treatments according to specific tumor characteristics [109].

In addition, most reported systems remain at the cellular or small-animal stage, and few have been evaluated using quantitative tissue oxygen mapping, clinically relevant pharmacokinetics, large-animal models, or long-term toxicology. This is primarily due to numerous barriers to clinical translation, including spatial and temporal oxygen heterogeneity, patient-to-patient and intratumoral reductase heterogeneity, activation in physiologically low-oxygen tissues, fluorescence attenuation, limited penetration depth, metabolic instability, differences between fluorophore and drug clearance, nanocarrier reproducibility, immunogenicity, and regulatory complexity. Therefore, current systems should be regarded primarily as preclinical proof-of-concept platforms.

### 6.6. Expanding Applications Beyond Oncology

Although most current research has focused on cancer therapy, hypoxia also plays a critical role in many other pathological processes, including ischemic diseases, inflammatory disorders, and microbial infections. Developing hypoxia-responsive fluorescent systems for these diseases may open new avenues for diagnosis and therapy. For example, hypoxia-responsive imaging probes could be used to visualize ischemic tissues in cardiovascular diseases or to monitor inflammatory responses under chronic inflammatory conditions. Similarly, hypoxia-activated antibacterial agents may provide more targeted therapeutic strategies for infections occurring in oxygen-deficient environments.

Overall, sustained interdisciplinary collaboration among chemists, biologists, and clinical researchers will be essential for advancing hypoxia-responsive fluorescent prodrugs and translating these systems into practical biomedical applications.

## 7. Conclusions

Hypoxia is a characteristic feature of many solid tumors and plays a critical role in tumor progression, metastasis, and therapeutic resistance. In recent years, hypoxia-responsive fluorescent prodrugs have emerged as a promising strategy for integrating molecular imaging with targeted therapy. By incorporating hypoxia-sensitive trigger groups, fluorescent reporters, and therapeutic agents into a single molecular platform, these systems enable selective drug activation in hypoxic tissues while allowing real-time visualization of the therapeutic process.

In this review, we summarize recent advances in the design and development of hypoxia-responsive fluorescent prodrugs. First, we briefly introduce the biological basis of pathological hypoxia and its important role in disease progression, highlighting the significance of targeting the hypoxic microenvironment. We then discuss common molecular design strategies for constructing hypoxia-responsive fluorescent prodrugs, including the introduction of bioreductive trigger groups such as nitroaromatic moieties, azo linkers, and N-oxide groups. These trigger groups can undergo selective reduction under hypoxic conditions, leading to linker cleavage, drug activation/release, or fluorescence activation. We also review the major fluorescence signaling mechanisms commonly employed in these systems, including PeT, FRET, ICT, and AIE mechanisms. These photophysical mechanisms enable sensitive and selective modulation of fluorescence signals, allowing researchers to visualize prodrug activation/release in living cells and tumor tissues. In addition, representative hypoxia-responsive fluorescent prodrug systems are summarized to illustrate recent progress in this field, with particular emphasis on their biomedical applications, including tumor hypoxia imaging, real-time monitoring of drug activation, and combination therapy. These systems show significant advantages in improving therapeutic selectivity and provide valuable information for understanding drug delivery and activation processes.

Despite encouraging progress, several challenges remain before hypoxia-responsive fluorescent prodrugs can be widely translated into clinical applications. Issues such as the spatial heterogeneity of tumor hypoxia, limited tissue penetration of fluorescence imaging, potential off-target activation, and various barriers to clinical translation from basic research still need to be addressed. Future studies should focus on developing more efficient hypoxia-responsive trigger groups, optimizing the optical properties of fluorophores, and integrating multimodal imaging strategies to improve diagnostic accuracy and therapeutic efficacy.

Overall, hypoxia-responsive fluorescent prodrugs represent a rapidly evolving research field at the interface of chemical biology, medicinal chemistry, and biomedical imaging. With continued advances in molecular design, imaging technologies, and translational research, these systems are expected to be further developed and ultimately contribute to more precise and effective therapeutic strategies for hypoxia-associated diseases.

## Figures and Tables

**Figure 1:**
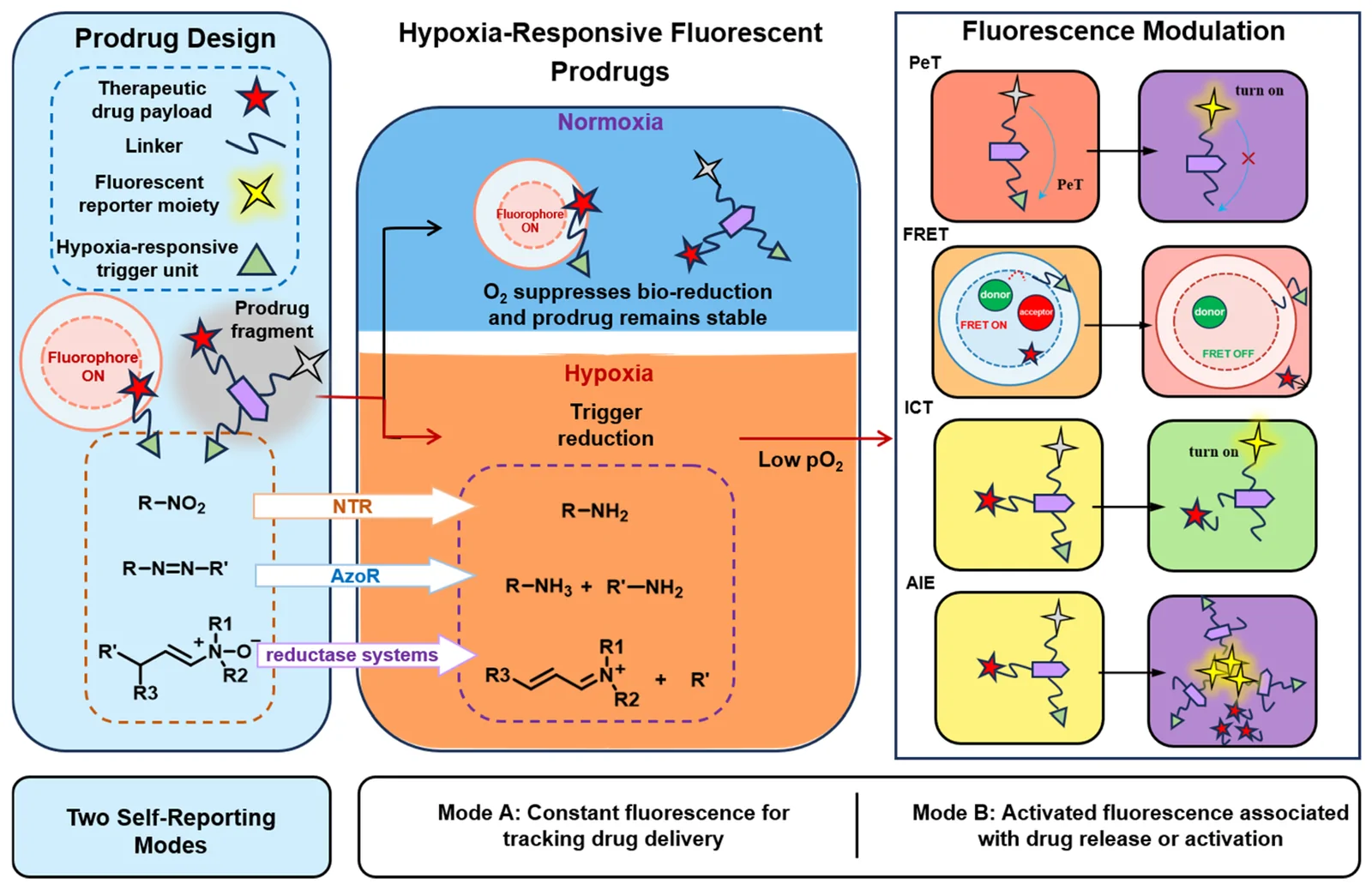
Design strategies and fluorescence modulation mechanisms of hypoxia-responsive fluorescent prodrugs: PeT, FRET, ICT, and AIE mechanisms.

**Figure 2:**
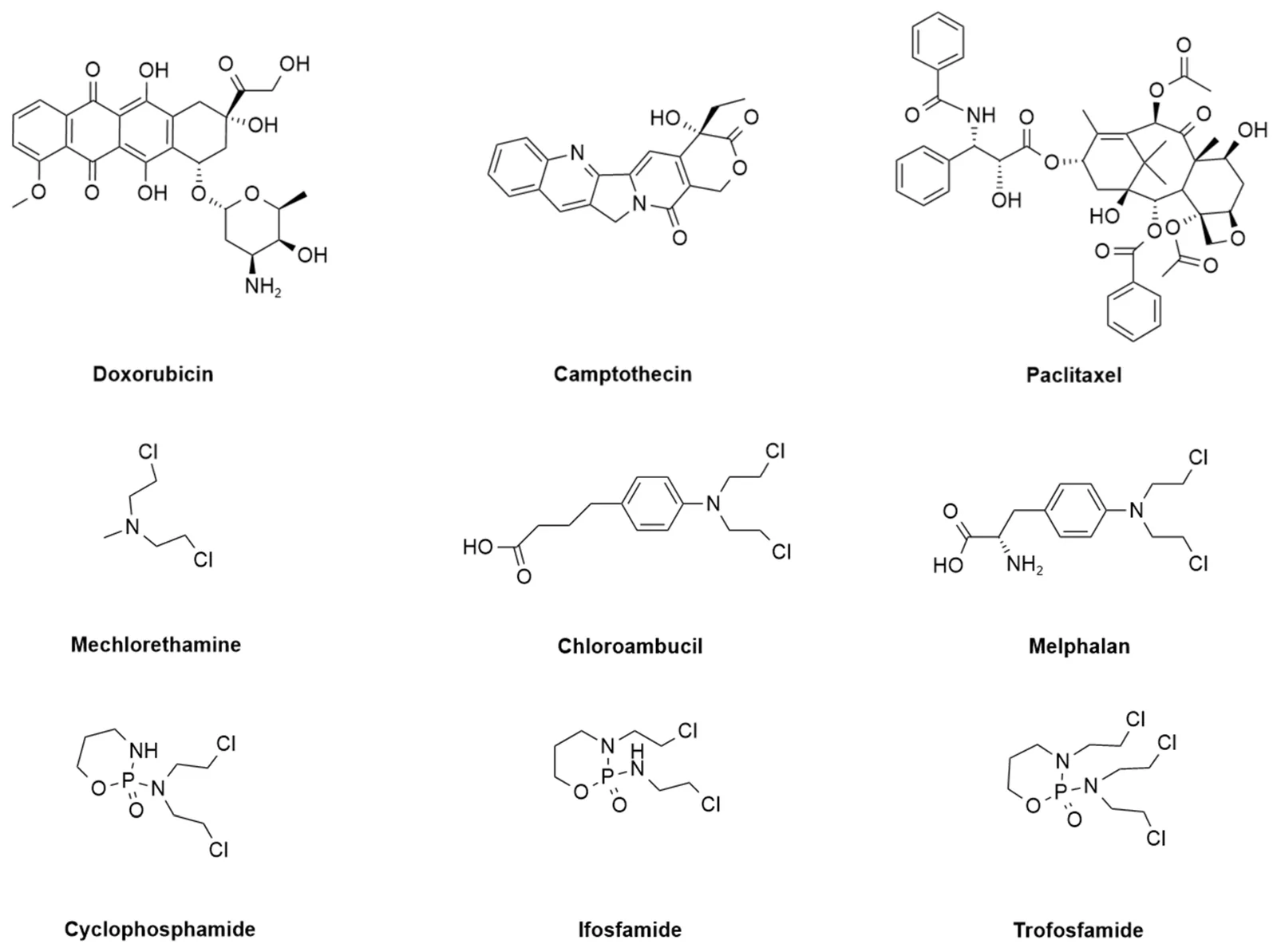
Chemical structure of some typical therapeutic drug payloads: doxorubicin, camptothecin, paclitaxel, and nitrogen mustard derivatives.

**Figure 3:**
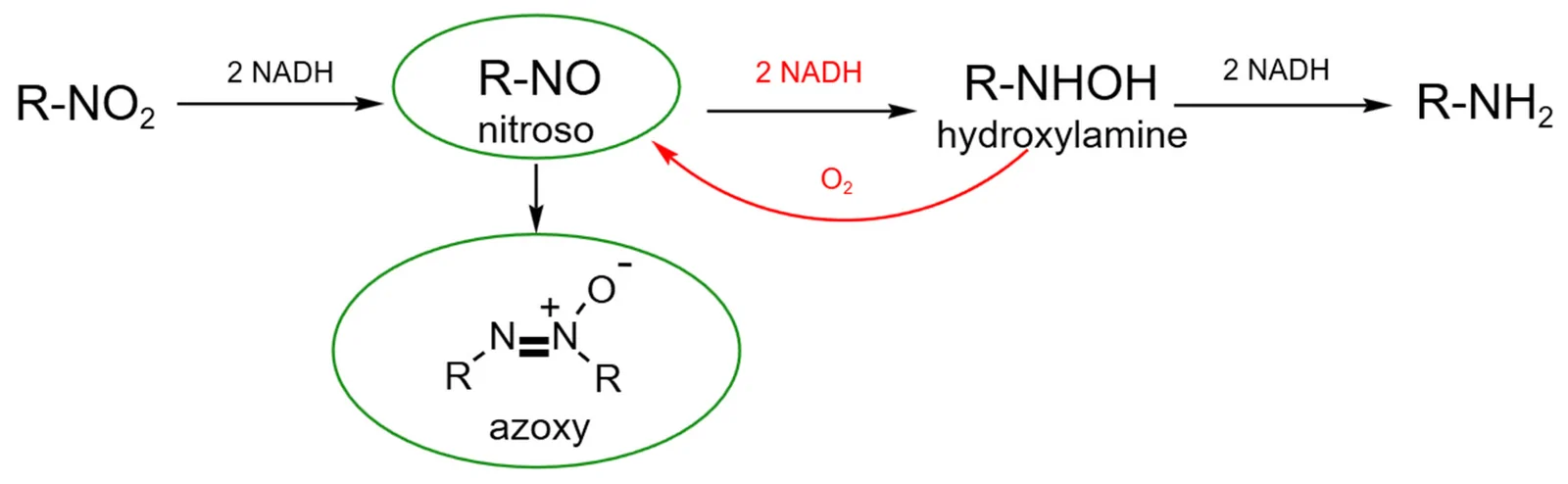
Mechanism illustration of NTR-catalyzed reduction in nitroaromatic compounds. Reproduced from ref. [37]. Copyright (2021), with permission from Elsevier.

**Figure 4:**
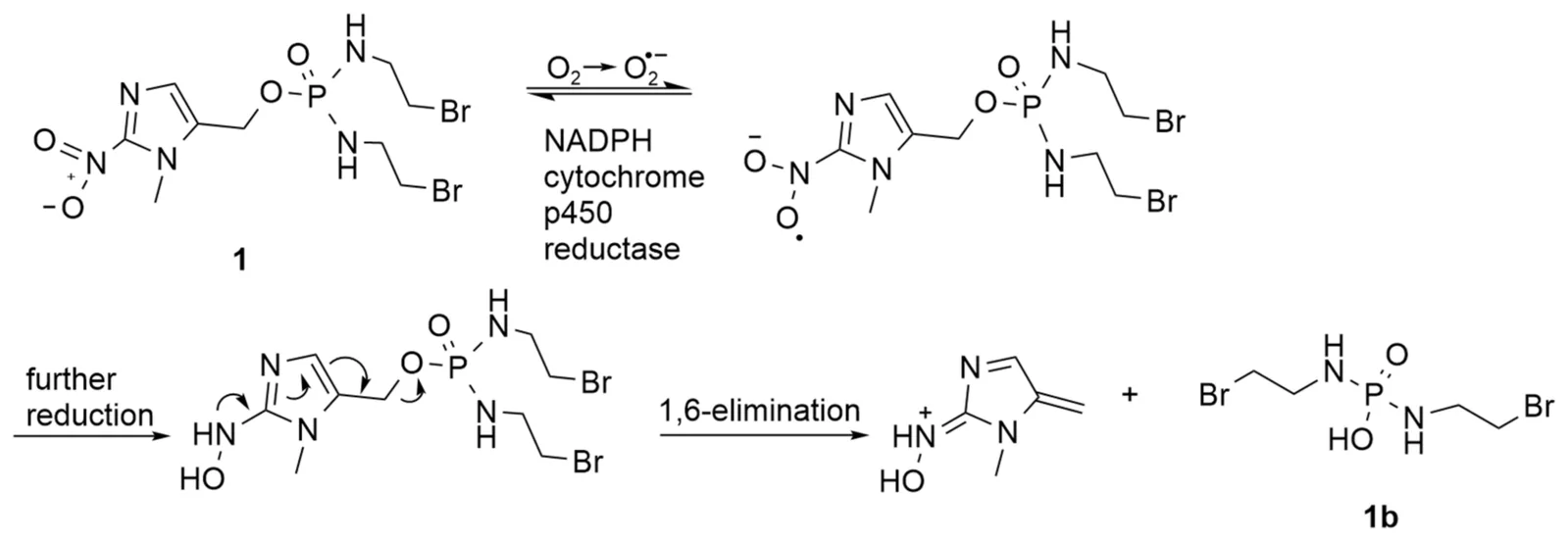
Postulated reduction pathway of **1** yielding the toxin (**1b**). Reproduced from ref. [39]. Copyright (2008), with permission from the American Chemical Society.

**Figure 5:**
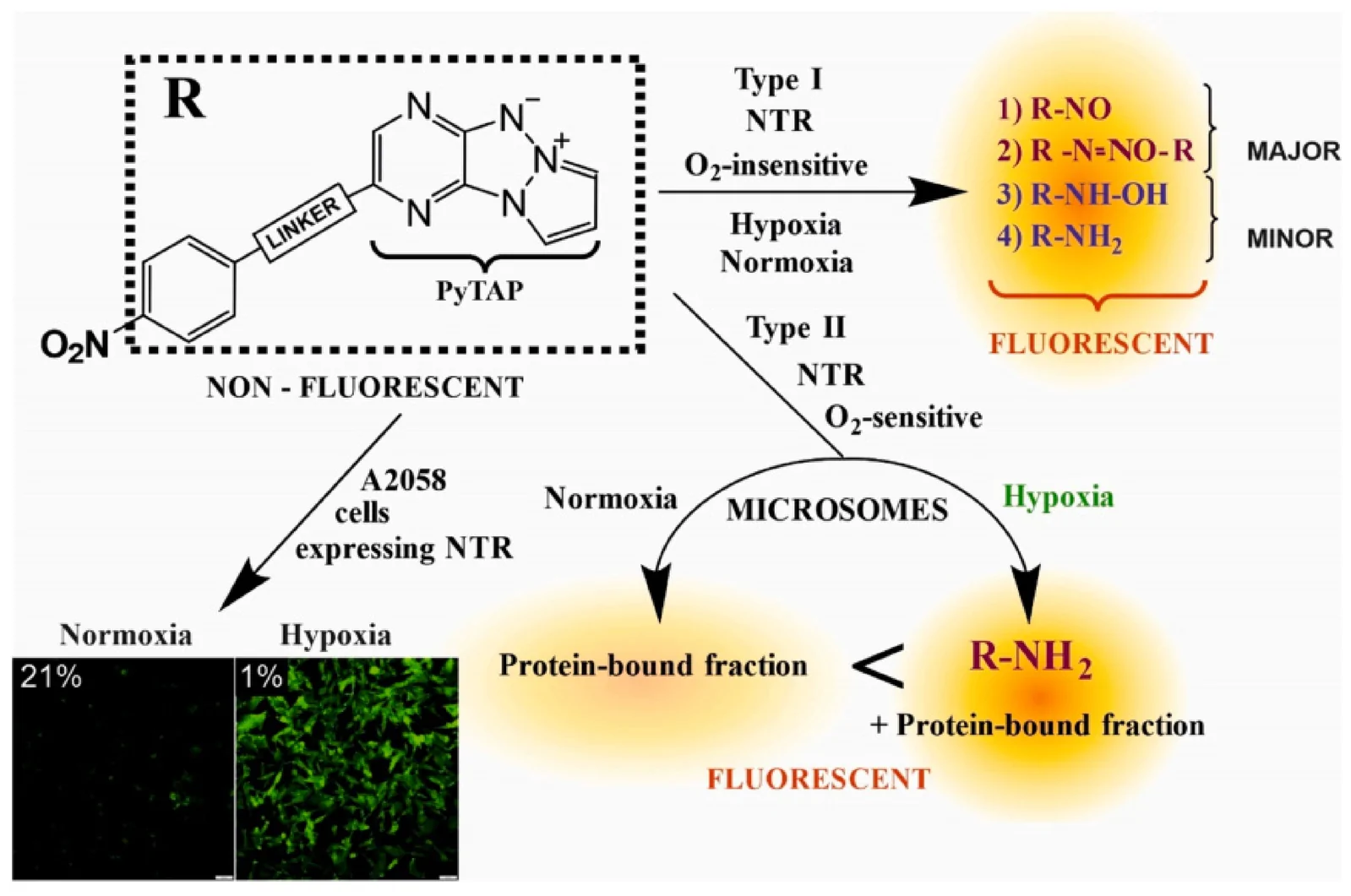
Schematic representation of the major outcomes of the investigated probes. Reproduced from ref. [37]. Copyright (2021), with permission from Elsevier.

**Figure 6:**
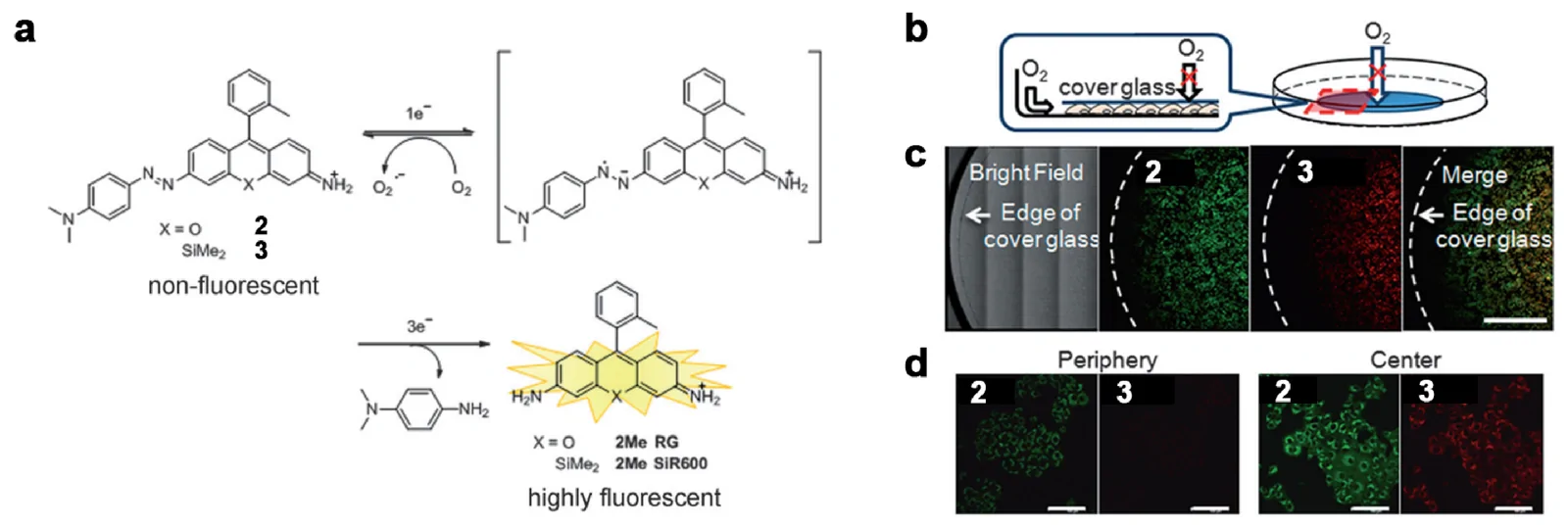
(**a**) Design strategy, chemical structure, and proposed detection mechanism for azo rhodamines (**2** and **3**). (**b**) Detection of a gradient of cellular hypoxia generated by placing a cover glass on top of the cells. (**c**) Bright-field and fluorescence confocal microscopy images of A549 cells onto which the cover glass was placed. Cells were incubated under normal culture conditions with the cover glass for 3 h in the presence of **2** (0.5 μM) and **3** (1 μM) containing DMSO (0.15%). Twenty images were combined into one image with the Leica SP5 software. Scale bar: 2.5 mm. (**d**) Magnified images of A549 cells near the boundary of the cover glass (periphery) and in the central part of the cover glass (center). Scale bar: 100 μm. The excitation and emission wavelengths were 488 and 515–553 nm for **2**, and 600 and 620–700 nm for **3**, respectively. Reproduced from ref. [43]. Copyright (2013), with permission from Wiley-VCH.

**Figure 7:**
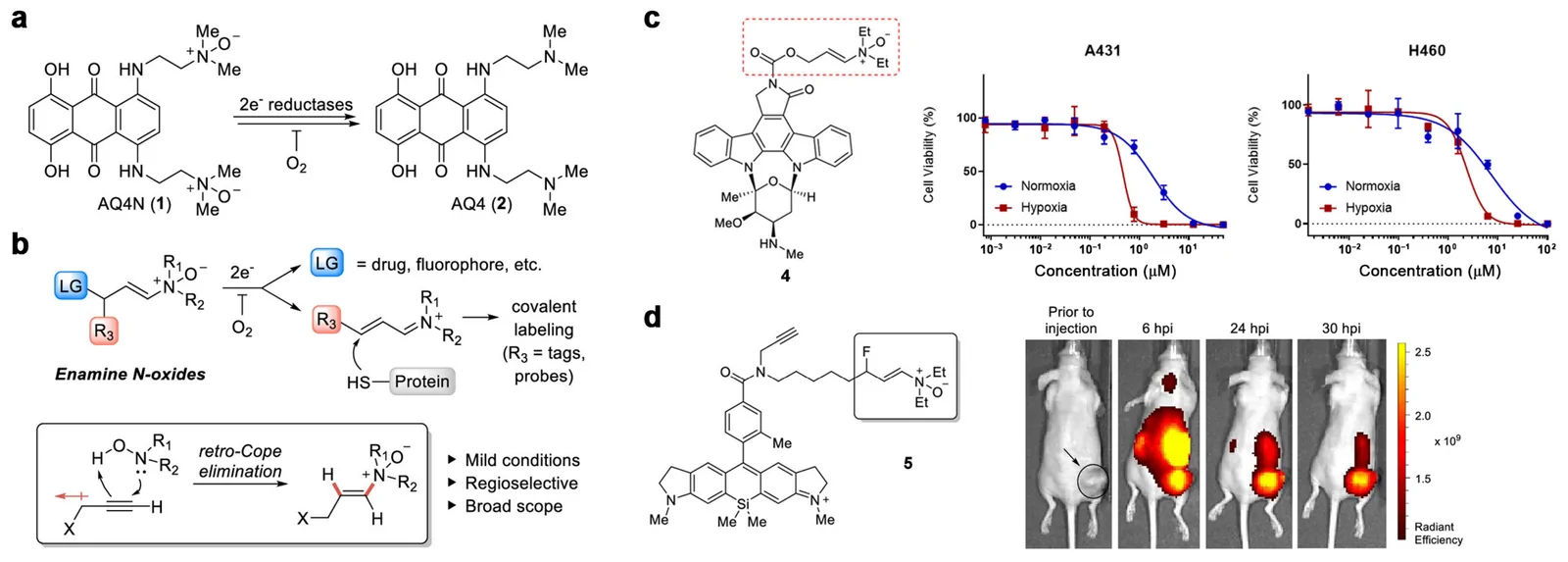
(**a**) Two sequential 2e^−^ reductions by hemeproteins convert aliphatic N-oxide prodrug AQ4N into the cytotoxic agent AQ4. (**b**) Design of new hypoxia-activated prodrugs termed enamine N-oxides. Enamine N-oxides can release small molecules upon 2e^−^ bioreduction selectively under hypoxic conditions, and Retro-Cope elimination between alkynes and dialkylhydroxylamine provides access to novel enamine N-oxides under mild reaction conditions. The resulting unsaturated iminium ion can readily react with biological nucleophiles. LG = leaving group. (**c**) Chemical structure of the hypoxia-responsive prodrug **4** and the dose-response cell viability curves for prodrug **4** in A431 and H460 cell lines. (**d**) Structure of alkyne-containing enamine N-oxide fluorescent probe **5** and fluorescence imaging of a BxPC-3 xenograft mouse model using **5**. Reproduced from ref. [48]. Copyright (2021), with permission from American Chemical Society.

**Figure 8:**
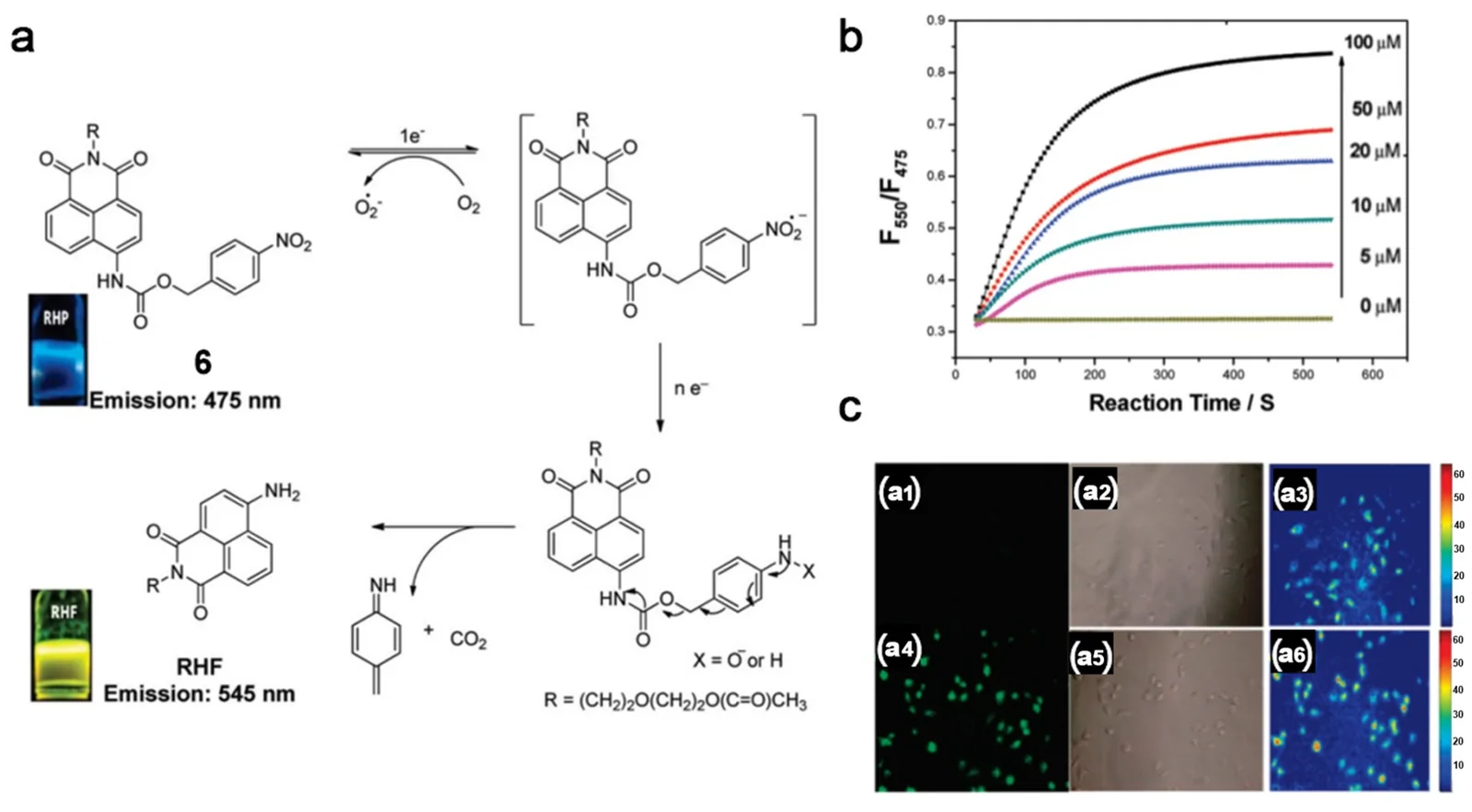
(**a**) Proposed detection mechanism of **6**. (**b**) Ratio changes (F_550_/F_475_) of **6** at varied concentrations over an incubation time of 10 min at 37 °C with 10 μg/mL NTR and 500 μM of NADH. λ_ex_ = 410 nm. (**c**) **6** was applied to hypoxia imaging in A549 tumor cells. Reproduced from ref. [54]. Copyright (2011), with permission from the American Chemical Society.

**Figure 9:**
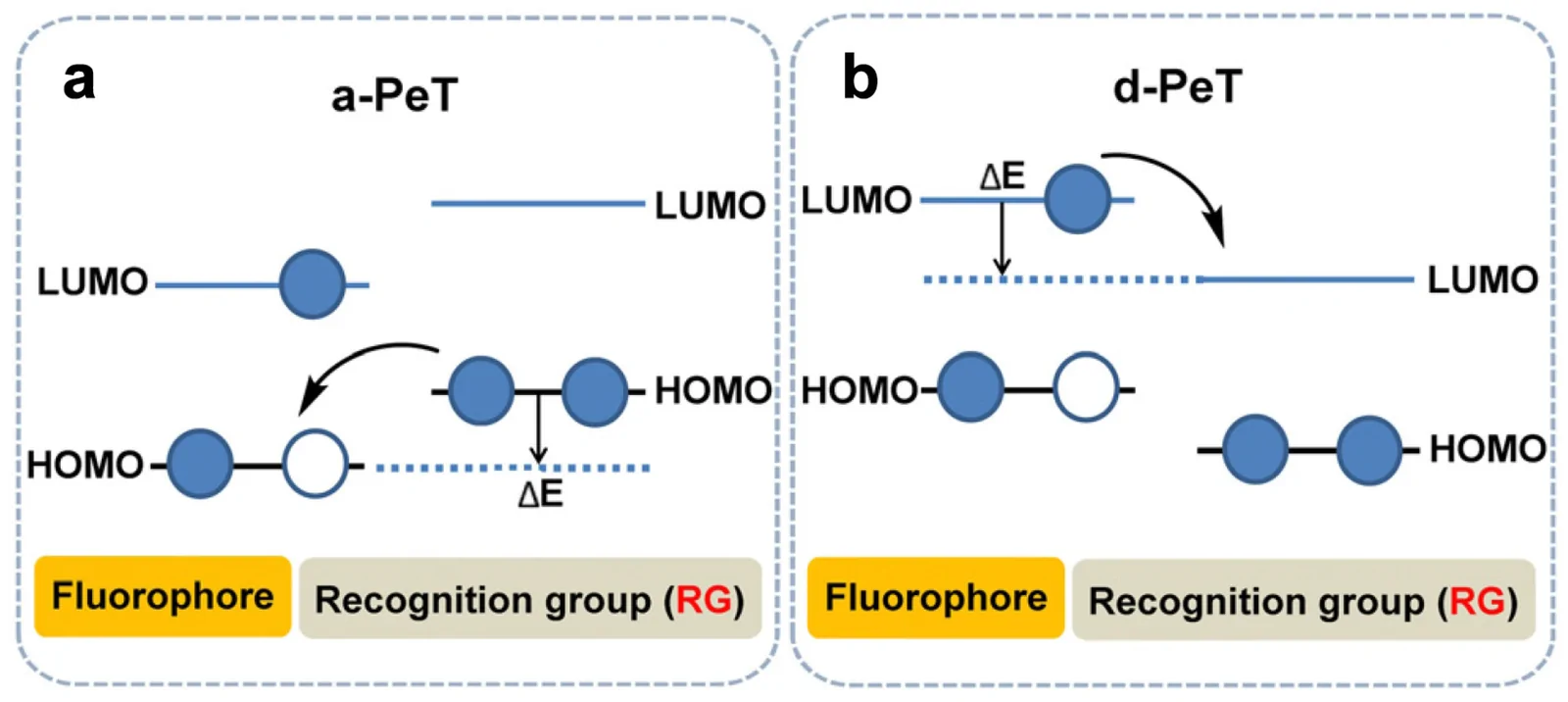
Modified frontier orbital energy diagram of PeT mechanism including a-PeT (**a**) and d-PeT (**b**). Reproduced from ref. [60]. Copyright (2023), with permission from the Royal Society of Chemistry.

**Figure 10:**
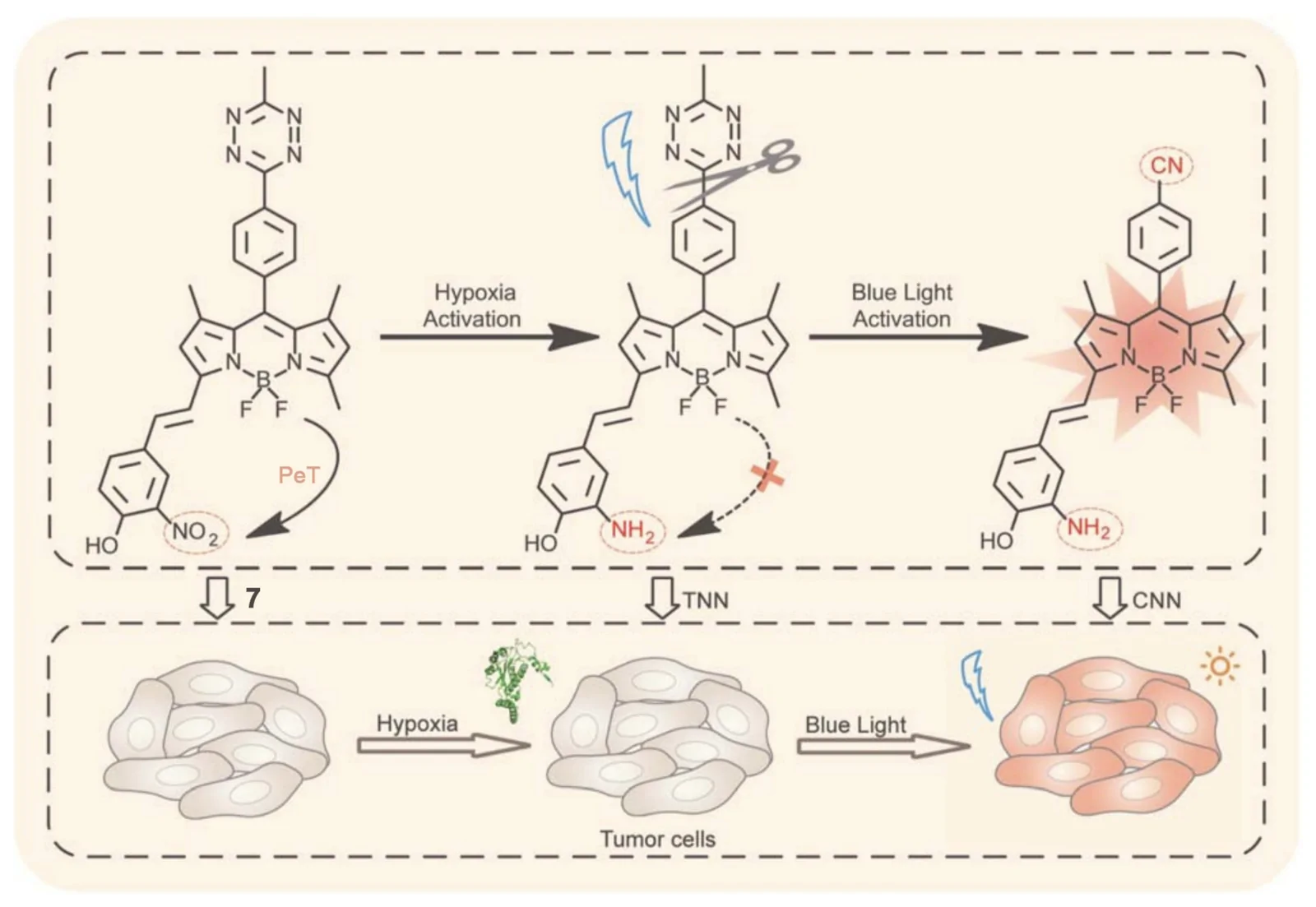
Illustrations of NTR and photoactivatable responsive hypoxic tumor cell imaging using the probe **7**. Reproduced from ref. [62]. Copyright (2022), with permission from the Royal Society of Chemistry.

**Figure 11:**
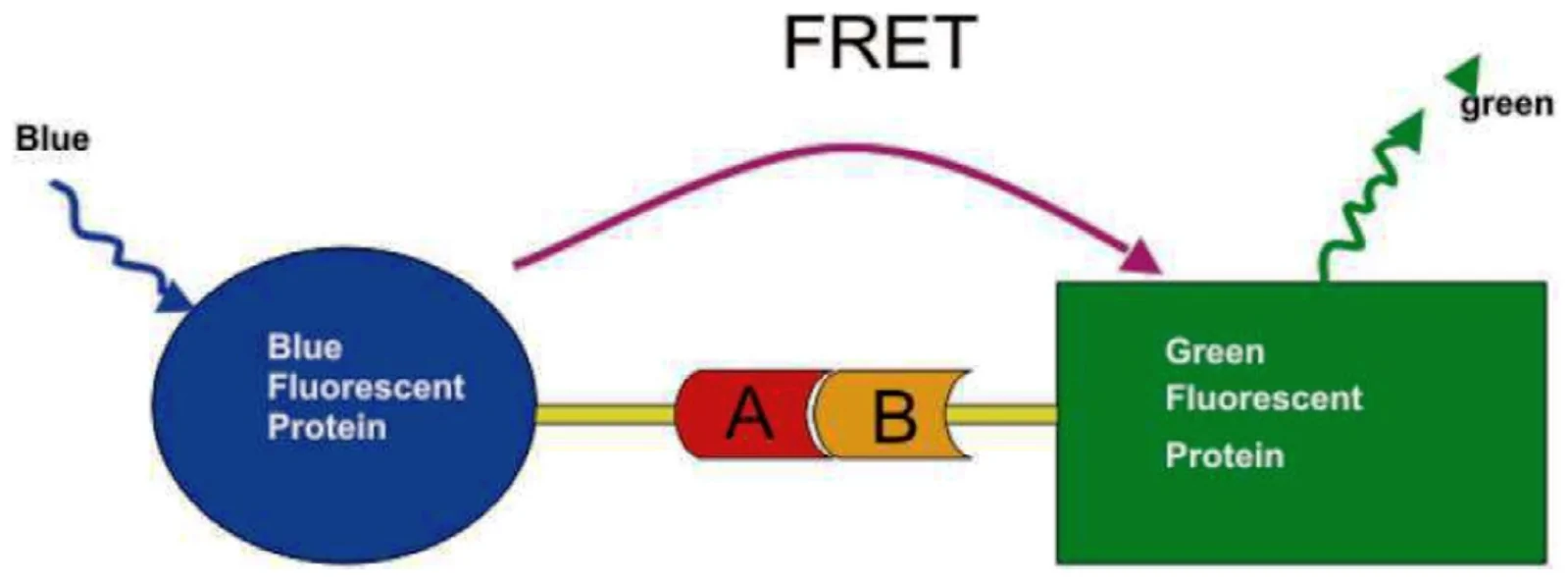
Schematic representation of the interaction of two different fluorescent proteins. Protein–protein interactions between proteins labeled A and B bring blue fluorescent protein and green fluorescent proteins in close enough proximity to allow for FRET to occur. In this example, excitation of the blue fluorescent protein results in the emission of fluorescence by the green fluorescent protein. Reproduced from ref. [63]. Copyright (2021), with permission from Elsevier.

**Figure 12:**
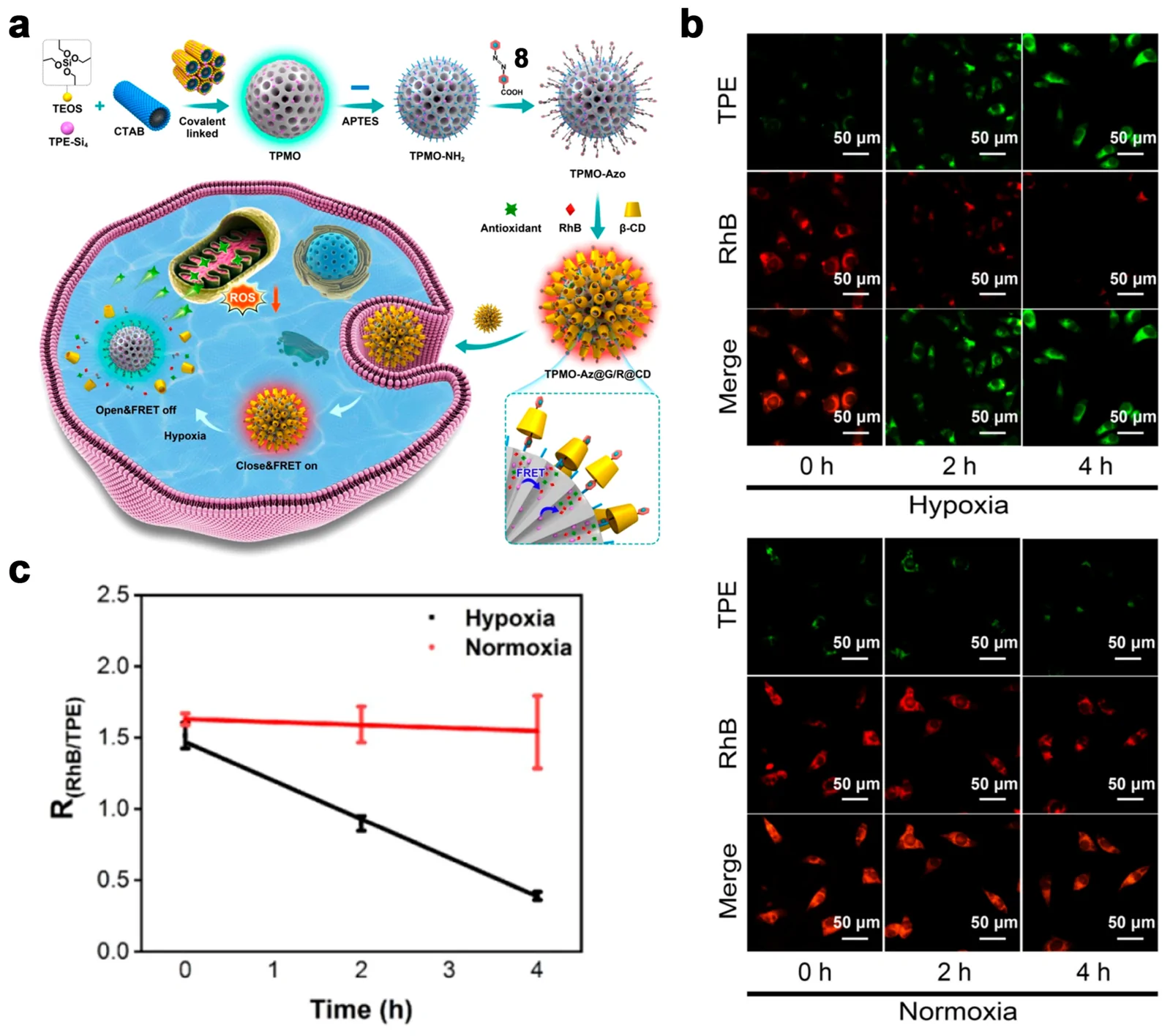
(**a**) Schematic illustration of TPMO-Az@G/R@CD for hypoxia-responsive cargo release, real-time imaging, and monitoring the extent of release in hypoxic cells. (**b**) Fluorescence inverted microscopy images of SH-SY5Y cells after treatment with TPMO-Az@G/R@CD for different times under hypoxia or normoxia conditions, respectively. Scale bar: 50 μm. (**c**) FRET ratio (RhB/TPE) recorded from SH-SY5Y cells cultured under normoxia and hypoxia conditions (*n* = 3). Reproduced from ref. [65]. Copyright (2021), with permission from the American Chemical Society.

**Figure 13:**
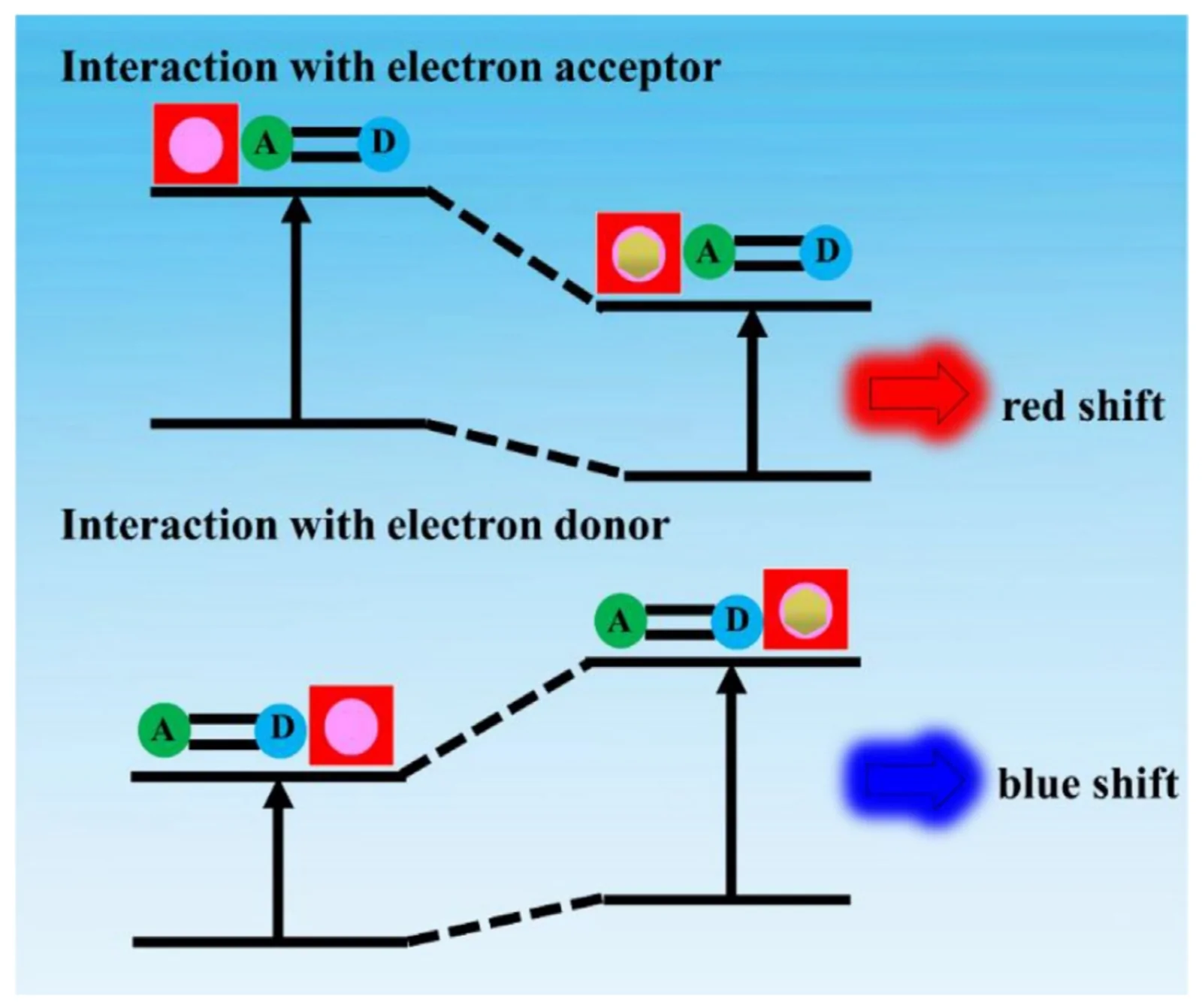
Recognition mechanism of ICT fluorescent probe. Reproduced from ref. [67]. Copyright (2023), with permission from Elsevier.

**Figure 14:**
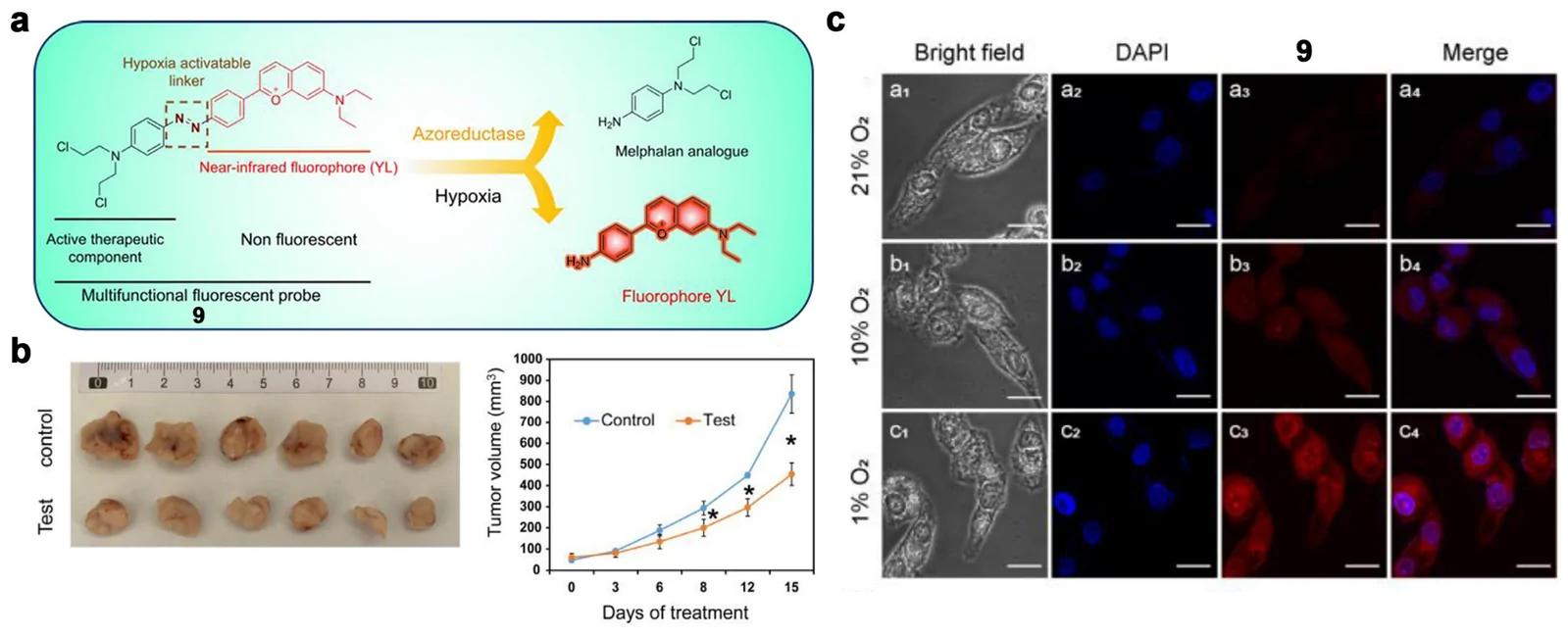
(**a**) Hypoxia fluorescent probe **9** and its activation. (**b**) Confocal fluorescence images of HeLa cells, cultured with different oxygen contents (1%, 10%, and 21%) for 10 h and incubated in PBS buffer solutions with **9** (5 μM) for 30 min before imaging. Scale bar: 20 μm. (**c**) In vivo therapeutic effects of **9**: Dissected tumor tissue images of mice treated with PBS (control) and **9** (1 mg/kg; test) in DMSO after 15 days, and tumor volume change in the two groups. * *p* < 0.05. Reproduced from ref. [69]. Copyright (2022), with permission from Elsevier.

**Figure 15:**
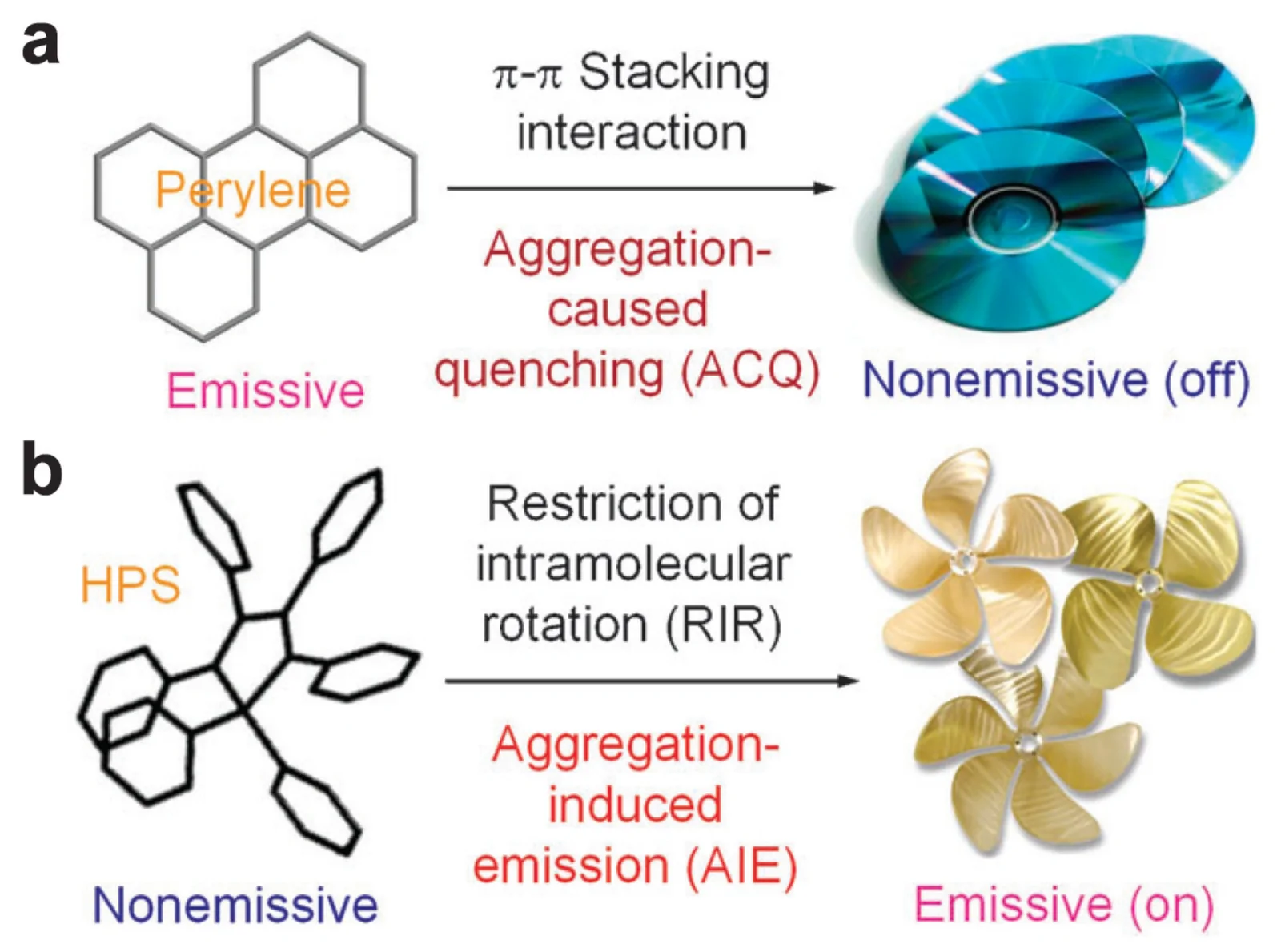
(**a**) Planar luminophores such as perylene tend to aggregate as disks pile up, due to the strong π–π stacking interactions between the aromatic rings, which commonly turns “off” light emission. (**b**) Non-planar luminogenic molecules such as hexaphenylsilole (HPS) behave oppositely, with their light emissions turned “on” by aggregate formation, due to the restriction of the intramolecular rotation (RIR) of the multiple phenyl rotors against the silole stator in the aggregate state. Reproduced from ref. [70]. Copyright (2011), with permission from the Royal Society of Chemistry.

**Figure 16:**
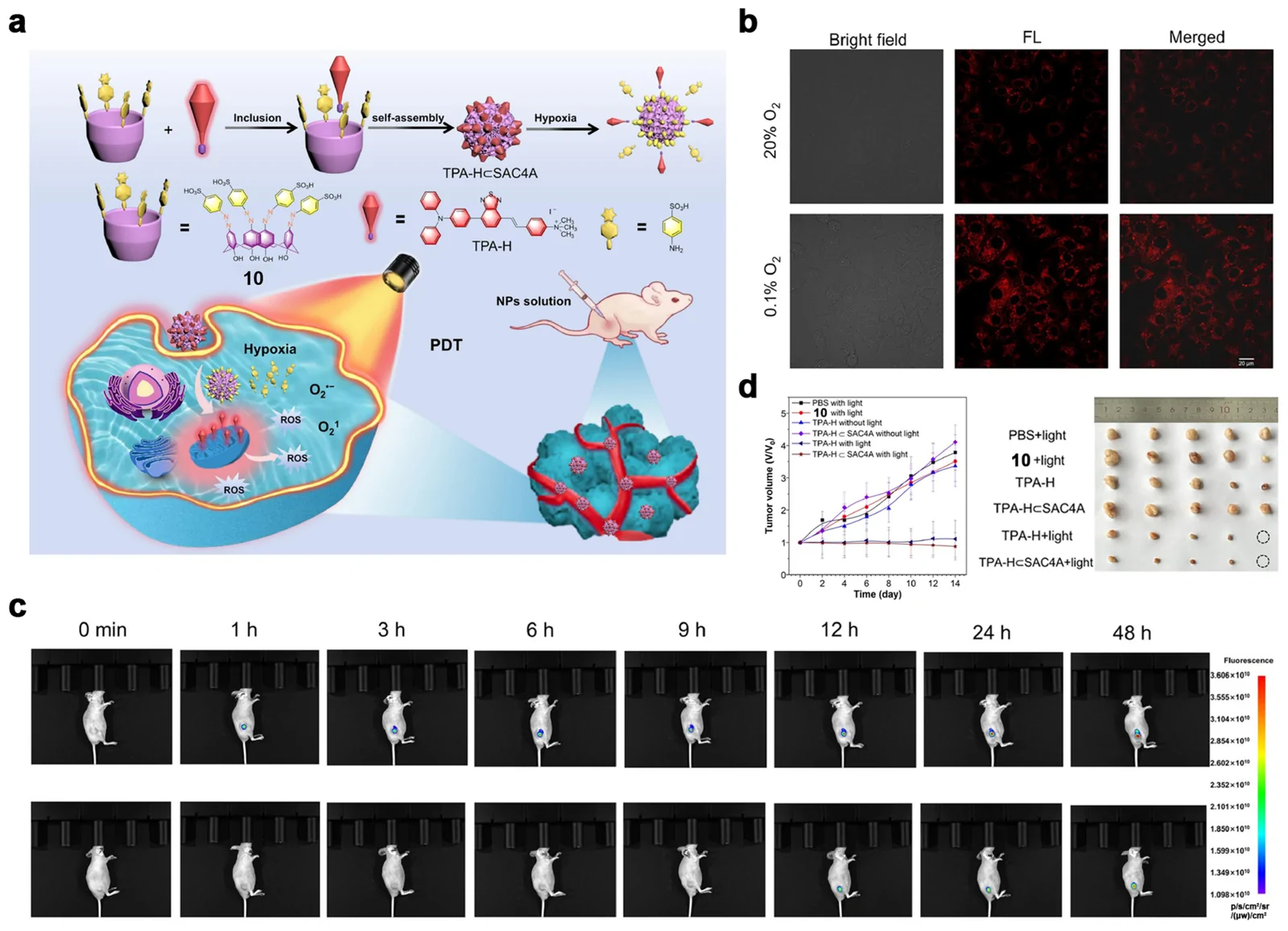
(**a**) Schematic illustration of the hypoxia-responsive supramolecular complexes **TPA-H⊂SAC4A**, and graphical diagram showing cell imaging and PDT of **TPA-H⊂SAC4A** complexes under hypoxia. (**b**) Confocal laser scanning microscopy images of HeLa cells incubated with **TPA-H⊂SAC4A** under hypoxic (less than 0.1% O_2_) or normoxic (20% O_2_) conditions for 12 h. λ_ex_ = 561 nm, λ_em_ = 600–700 nm. Scale bar = 20 μm. (**c**) Time-dependent in vivo images of HeLa tumor-bearing BALB/c nude mice injected in situ with TPA-H or **TPA-H⊂SAC4A**. (**d**) The tumor volume changes during the treatment process, and the corresponding tumor images in different treatment groups. Reproduced from ref. [72]. Copyright (2025), with permission from Science China Press.

**Figure 17:**
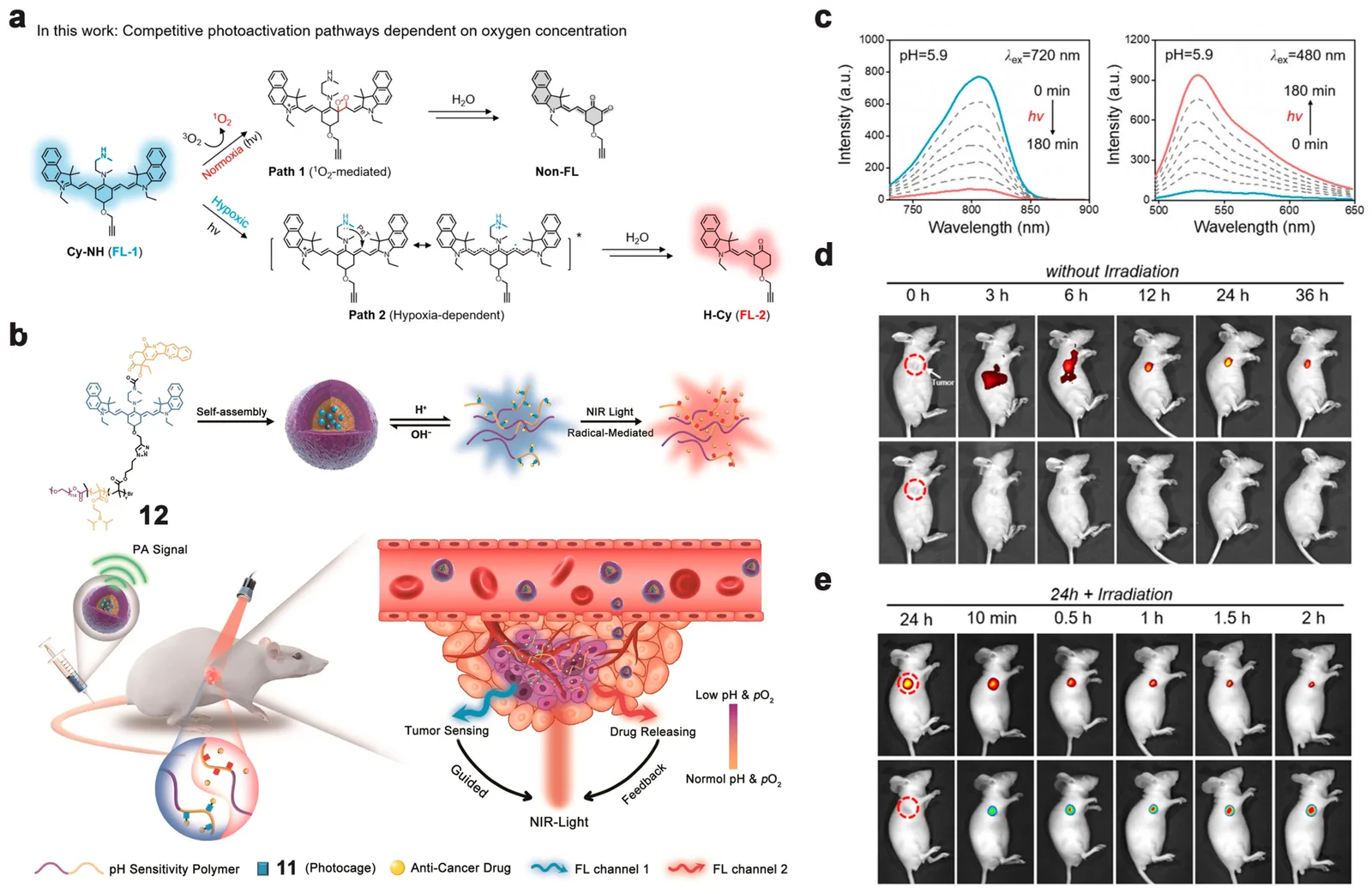
(**a**) Competitive photoactivation pathways dependent on oxygen concentration. Under light irradiation under hypoxia, free radicals are possibly generated from **11**, then result in the cleavage of **11**, and concurrently produce dual-channel fluorescence output. (**b**) Schematic illustration of photocage **12** with dual-channel fluorescence output and schematic diagram of NIR light-initiated tumor-targeted precision drug release. (**c**) Emission spectra of **12** at λ_ex_ = 720 nm and λ_ex_ = 480 nm after NIR light irradiation at pH 5.9. (**d**,**e**) In vivo dual-channel NIR-fluorescence imaging of A549 xenograft tumor-bearing mice at various times (0, 3, 6, 9, 12, 24 h or under irradiation 10 min, 0.5 h, 1 h, 1.5 h, and 2 h) after intravenous injection of **12**, (**d**) without or with (**e**) light irradiation, administered at a CPT-equivalent dose of 0.1 mg kg^−1^. Reproduced from ref. [74]. Copyright (2021), with permission from Wiley-VCH.

**Figure 18:**
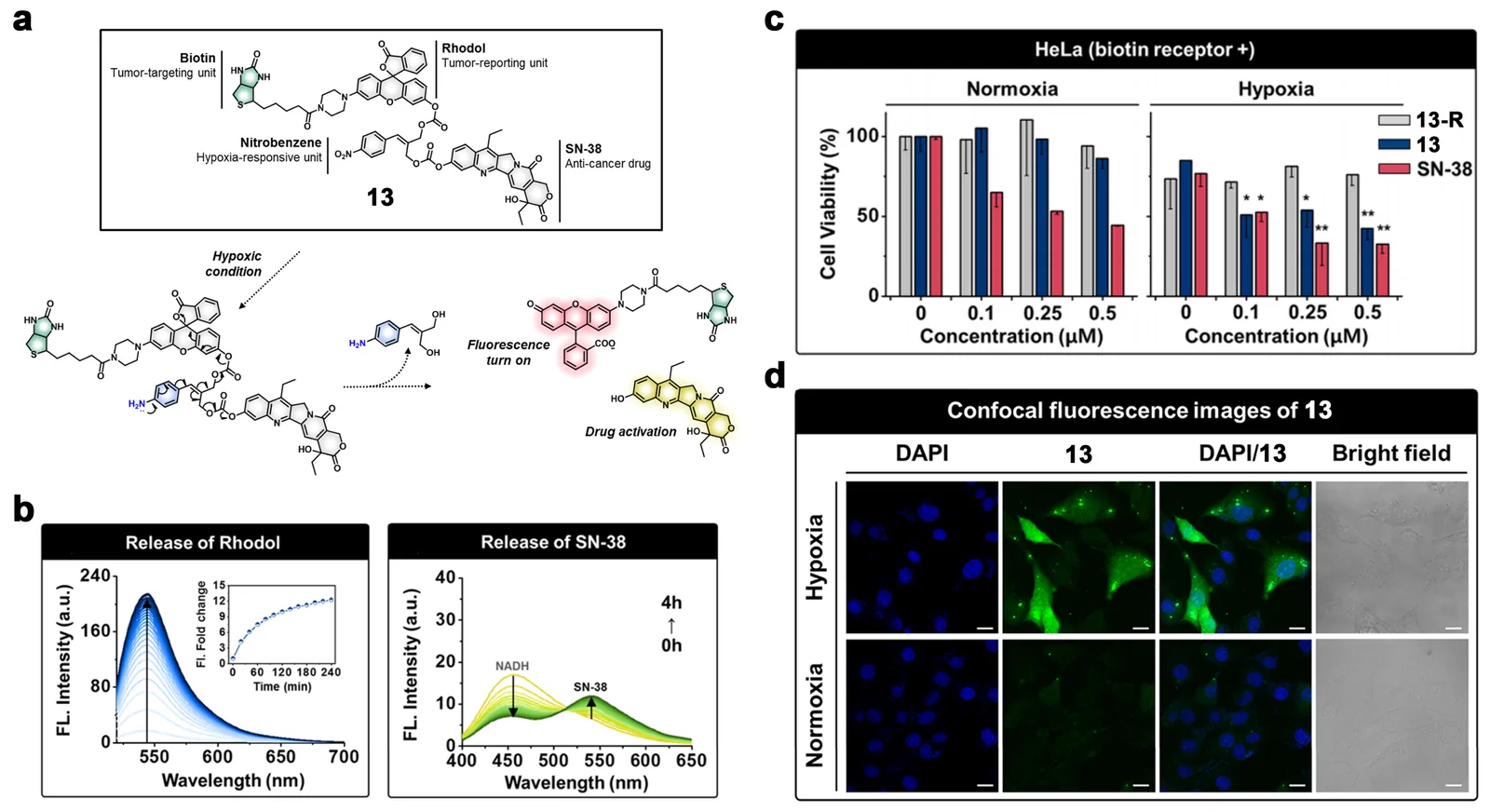
(**a**) Mechanism of drug release and fluorescence turn-on of theranostic, small-molecule-based prodrug **13** under hypoxic conditions. (**b**) Time-dependent fluorescence emission spectra of **13** following treatment with NTR and NADH, showing release of rhodol (λ_ex_ = 510 nm) and SN38, respectively. (**c**,**d**) Effect of prodrug **13**, reference compound 13-R, and SN-38 on cell viability of HeLa cells (**c**), and confocal fluorescence images of HeLa cells treated with 13 (green) and DAPI (blue) (**d**) under normoxic and hypoxic (0.1% O_2_) conditions. Scale bar: 20 μm. * *p* < 0.05, ** *p* < 0.01. Reproduced from ref. [78]. Copyright (2019), with permission from the American Chemical Society.

**Figure 19:**
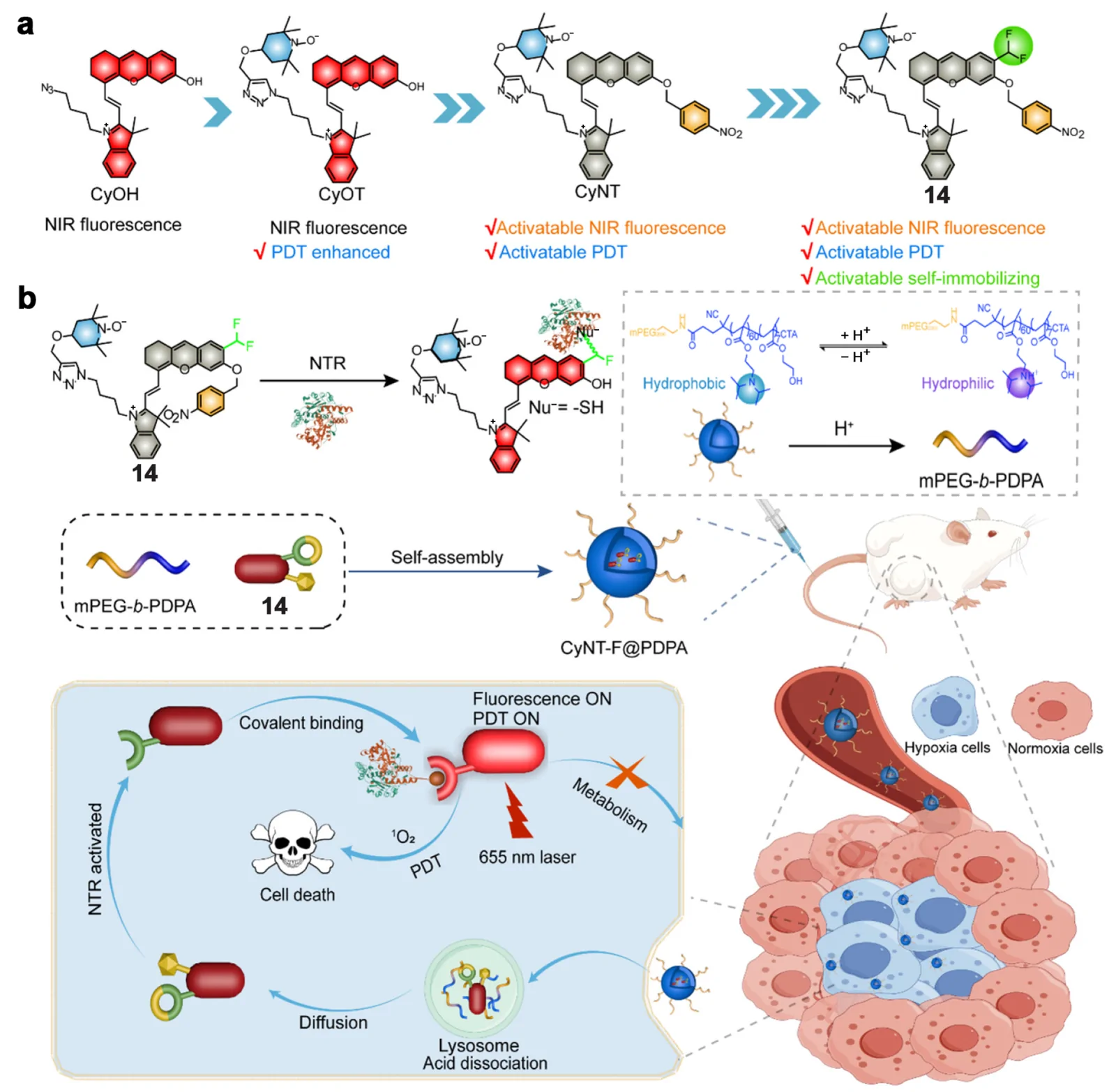
(**a**) Schematic illustration of the molecular design of the NTR-activatable and self-immobilizing photosensitizer **14**. (**b**) The mechanism of NTR-activated protein binding and micelle dissociation; schematic illustration of NTR-activated self-immobilizing photosensitizer to achieve photodynamic therapy of hypoxic tumors. Reproduced from ref. [79]. Copyright (2024), with permission from the American Chemical Society.

**Figure 20:**
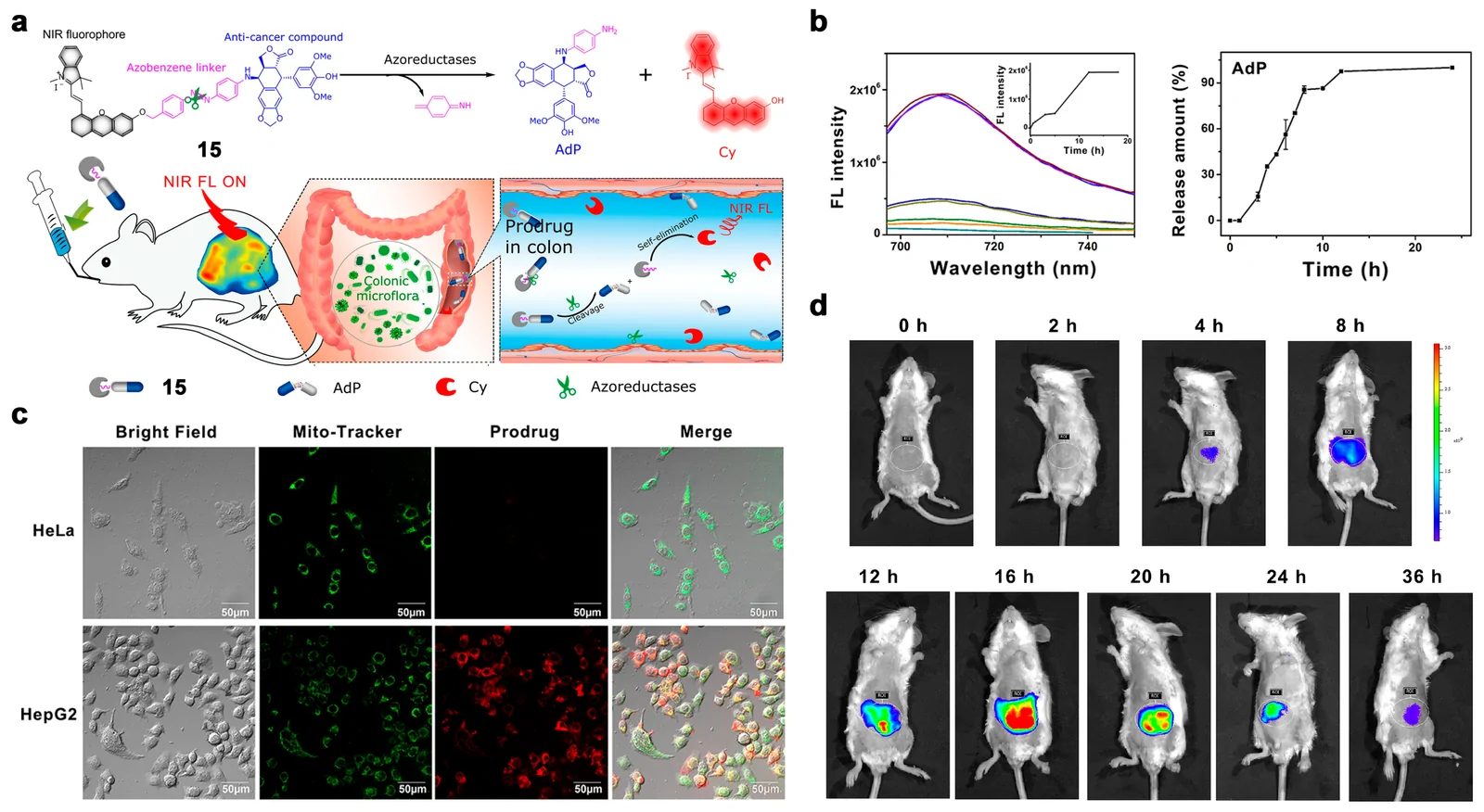
(**a**) Schematic illustration of targeted AdP release and NIR fluorescence emission “turn-on” in the colon from the proposed prodrug **15** upon reduction. (**b**) Time dependence of fluorescence intensity at 708 nm for **15** and AdP release percentage from **15** upon incubation with 1 mL of rat cecal contents. (**c**) Fluorescence imaging and colocalization study of HeLa and HepG2 cells after incubation with **15** and Mito-Tracker Green for 2 h. (**d**) In vivo fluorescence images of mice at various time points after gavage with **15** (0.1 mL, 2 mg kg^−1^). Reproduced from ref. [80]. Copyright (2020), with permission from the American Chemical Society.

**Figure 21:**
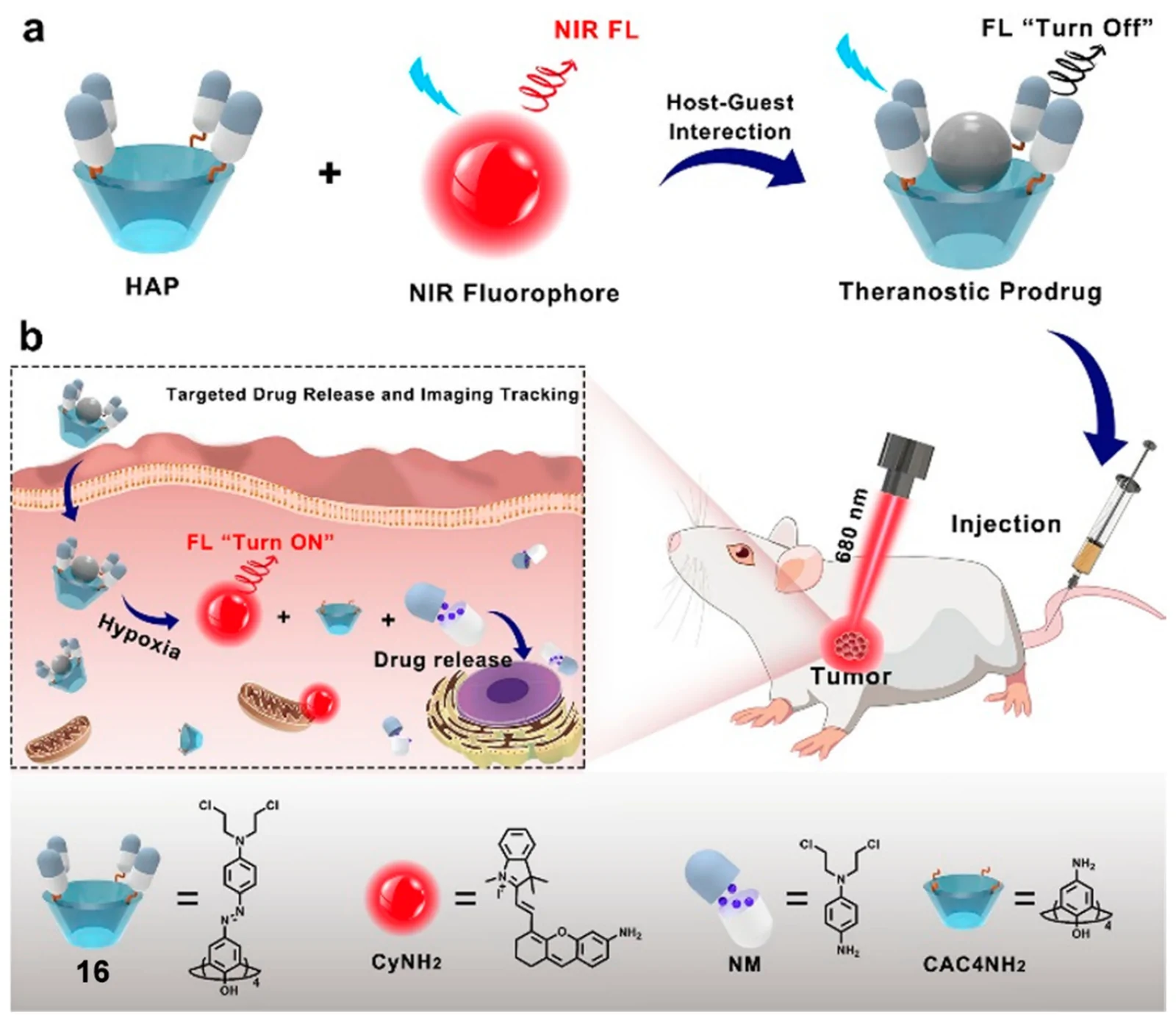
Schematic Illustration of the (**a**) proposed noncovalent strategy for the construction of a hypoxia-activated theranostic prodrug (**NMAC4A-CyNH_2_**) and (**b**) targeted NM release and NIR fluorescence “turn on” in a tumor hypoxic microenvironment. Reproduced from ref. [81]. Copyright (2021), with permission from the American Chemical Society.

**Figure 22:**
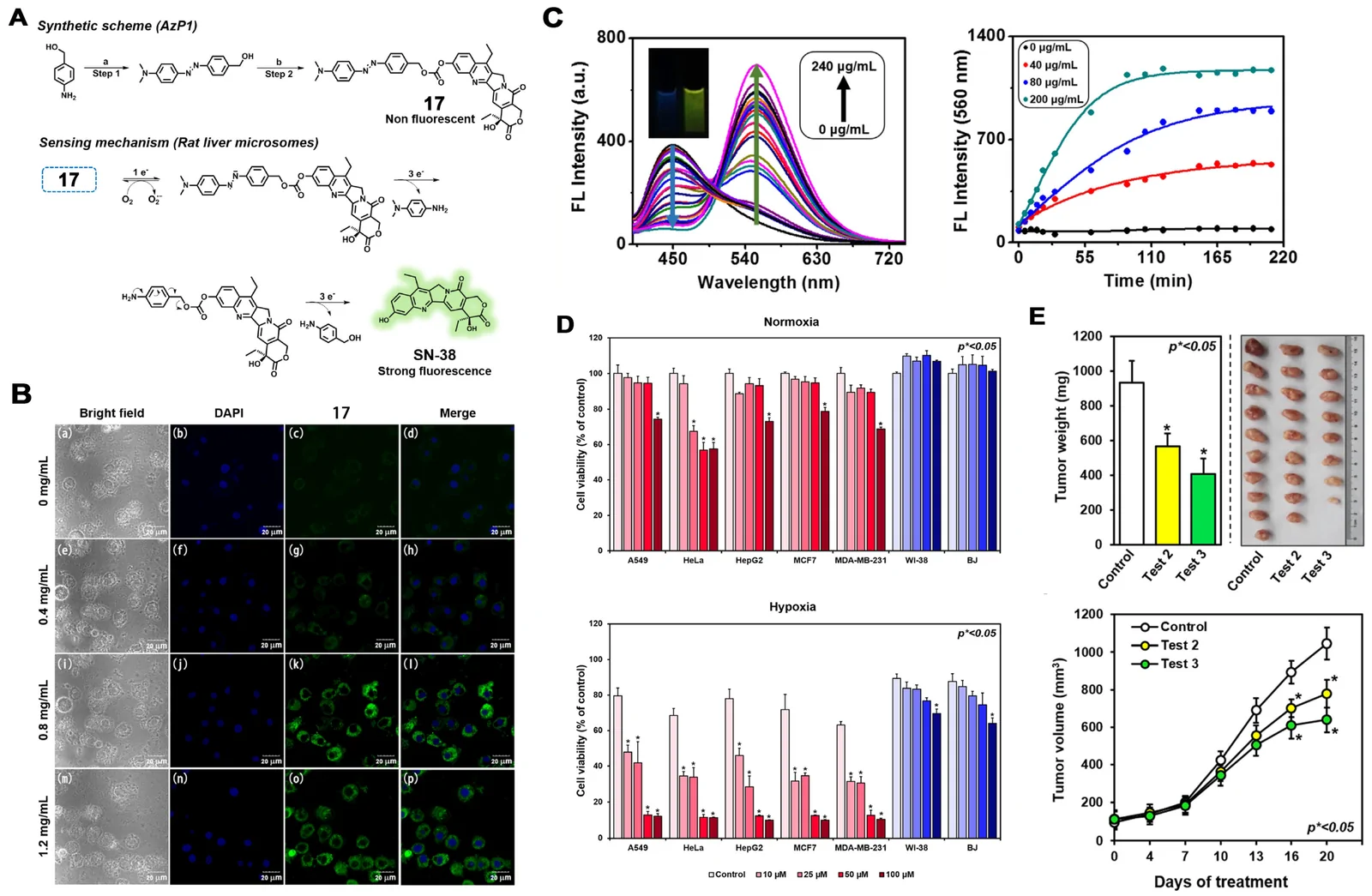
(**A**) The structure and synthetic route of theranostic prodrug **17** and the proposed sensing mechanism under hypoxic conditions. (**B**) Fluorescence emission spectrum and time-dependent fluorescence intensity at 560 nm of **17** (10 µM) in the presence of varying amounts of rat liver microsomes (0 µg/mL~240 µg/mL), containing 0.1 mM NADH as a coenzyme. (**C**) Confocal fluorescence images of living HeLa cells, incubated with **17** (10 µM) at different oxygen levels (with antioxidant at 0 µg/mL~1.2 µg/mL) for 2 h. (**D**) Cytotoxicity of compound **17** under normoxic and hypoxic conditions. (**E**) In vivo therapeutic effects of **17**: tumor tissue weights after treatment with PBS, 10 μM (Test 2), or 20 μM (Test 3) of **17** in 100 μL PBS, and tumor volume change in all three test groups. * *p* < 0.05. Reproduced from ref. [82]. Copyright (2018), with permission from Elsevier.

**Figure 23:**
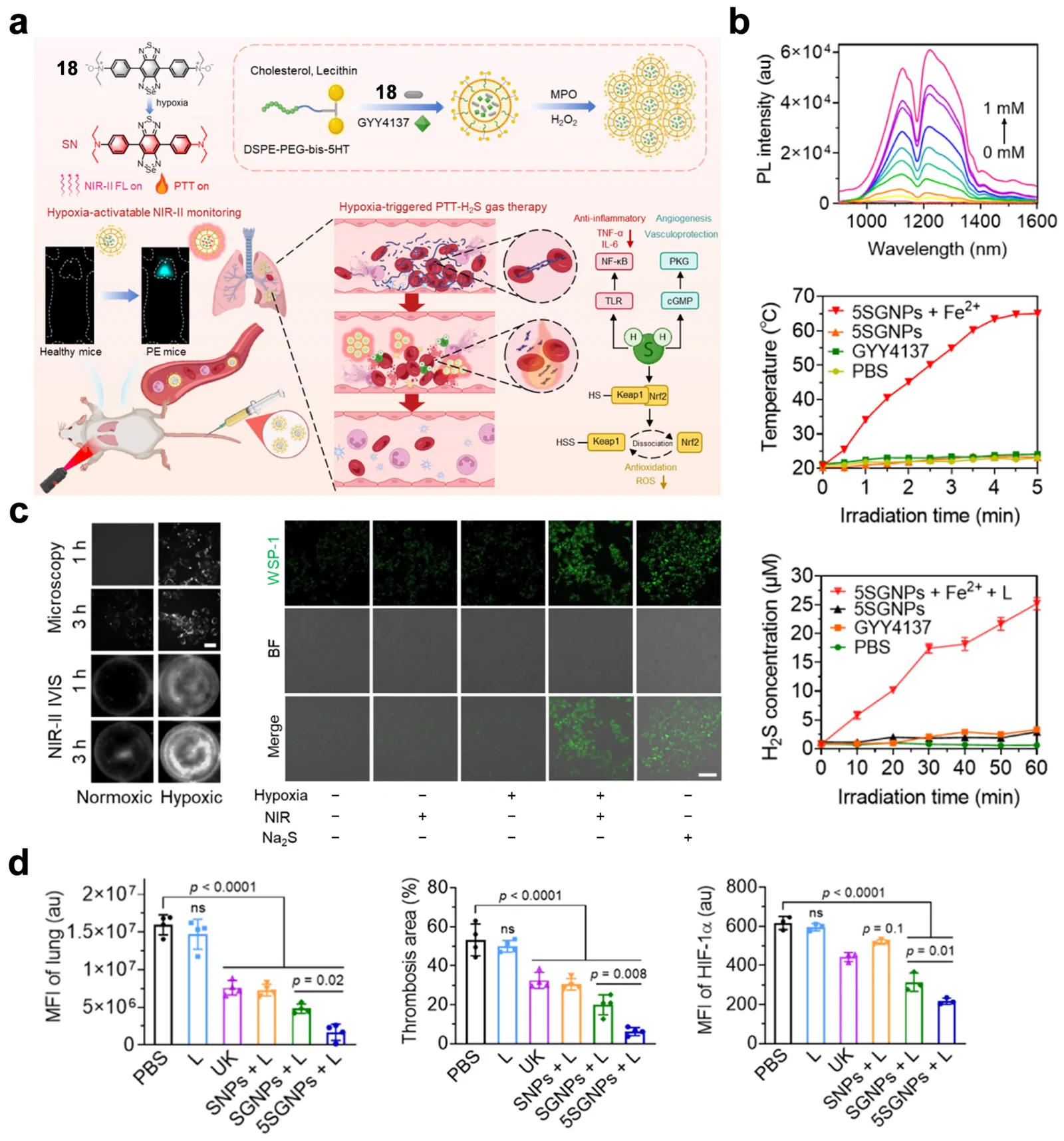
(**a**) Hypoxia-responsive molecular probe (**18**) and a H_2_S donor (GYY4137) were encapsulated into liposomes, which were further conjugated with bis-5HT to enhance the retention at thrombotic site. (**b**) PL spectra changes of **18**, with the treatment of different concentrations of Fe^2+^; temperature increase curves of different formulations and in situ H_2_S release capability of different formulations under 808 nm light irradiation. (**c**) NIR-II fluorescence activation of **5SGNPs** in normoxic or hypoxic HUVECs after 1 and 3 h incubation; confocal laser scanning microscopy images showing the intracellular H_2_S release from **5SGNPs** using WSP-1 as the indicator. Scale bar: 50 µm. (**d**) Thrombolytic efficacy of **5SGNPs** in PE mice evaluated by the FITC-fibrin fluorescence intensity in the lungs of mice, the percentages of thrombus area in pulmonary vessels, and HIF-1α fluorescence intensities in the lung following various treatments. ns, not significant. Reproduced from ref. [83]. Copyright (2025), with permission from the American Chemical Society.

**Figure 24:**
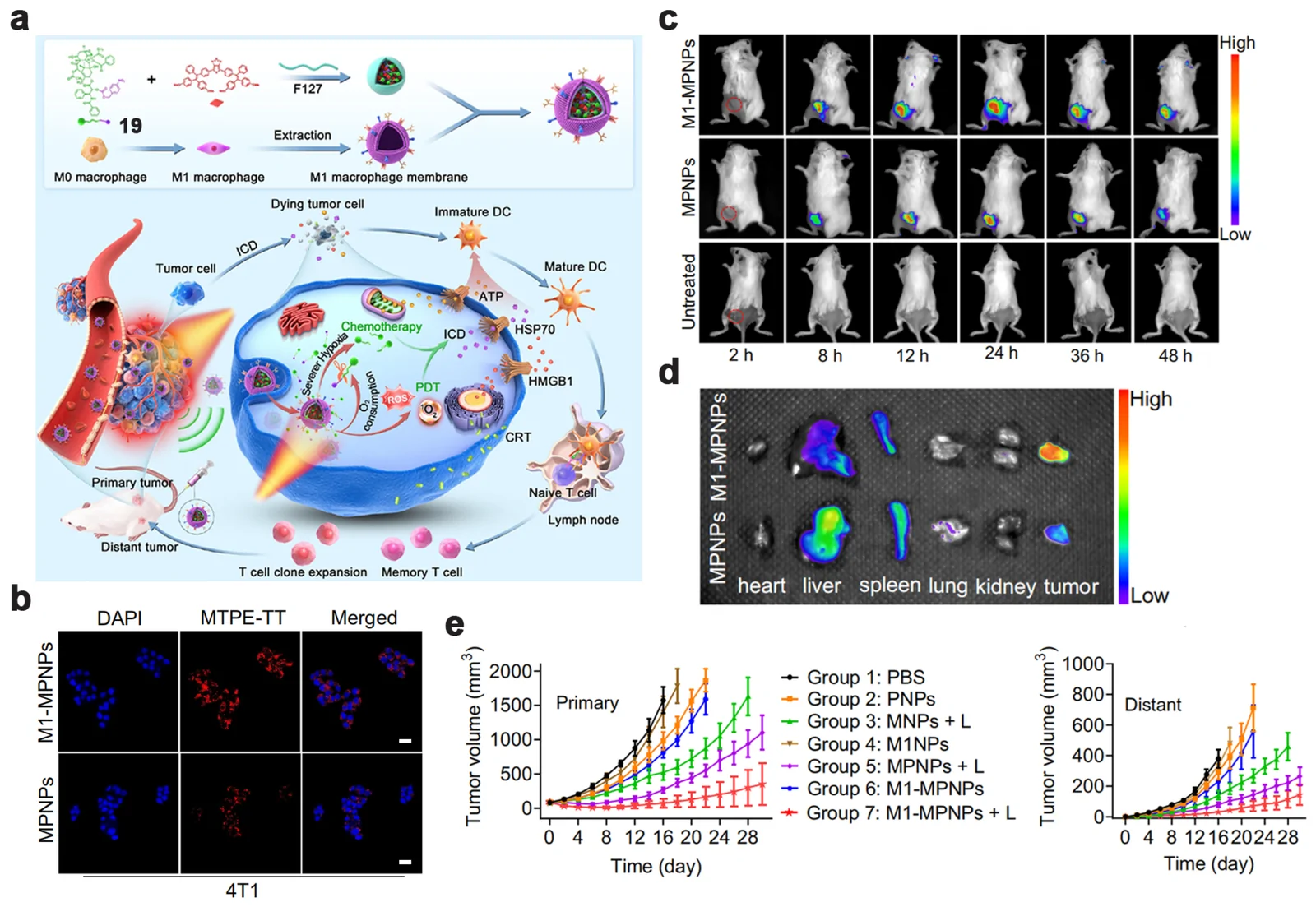
(**a**) Schematic illustration of the photo-triggered self-accelerated nanoplatform for fluorescence and PA imaging-guided combination immunotherapy. (**b**) Representative confocal laser scanning microscopy images of 4T1 cells upon incubation with **MPNPs** or **M1-MPNPs**. Scale bars: 20 µm. (**c**) Representative fluorescence images of 4T1 tumor-bearing mice at various time points post i.v. injection of **MPNPs** or **M1-MPNPs**; tumor-bearing mice with no treatment were used as controls. (**d**) Representative ex vivo fluorescence imaging of major organs and tumors isolated from mice at 24 h post i.v. injection of **MPNPs** or **M1-MPNPs**. (**e**) Plots of primary tumor volume and distant tumor volume in bilateral 4T1 tumor-bearing mice post various treatments. Reproduced from ref. [84]. Copyright (2023), with permission from Springer Nature.

**Figure 25:**
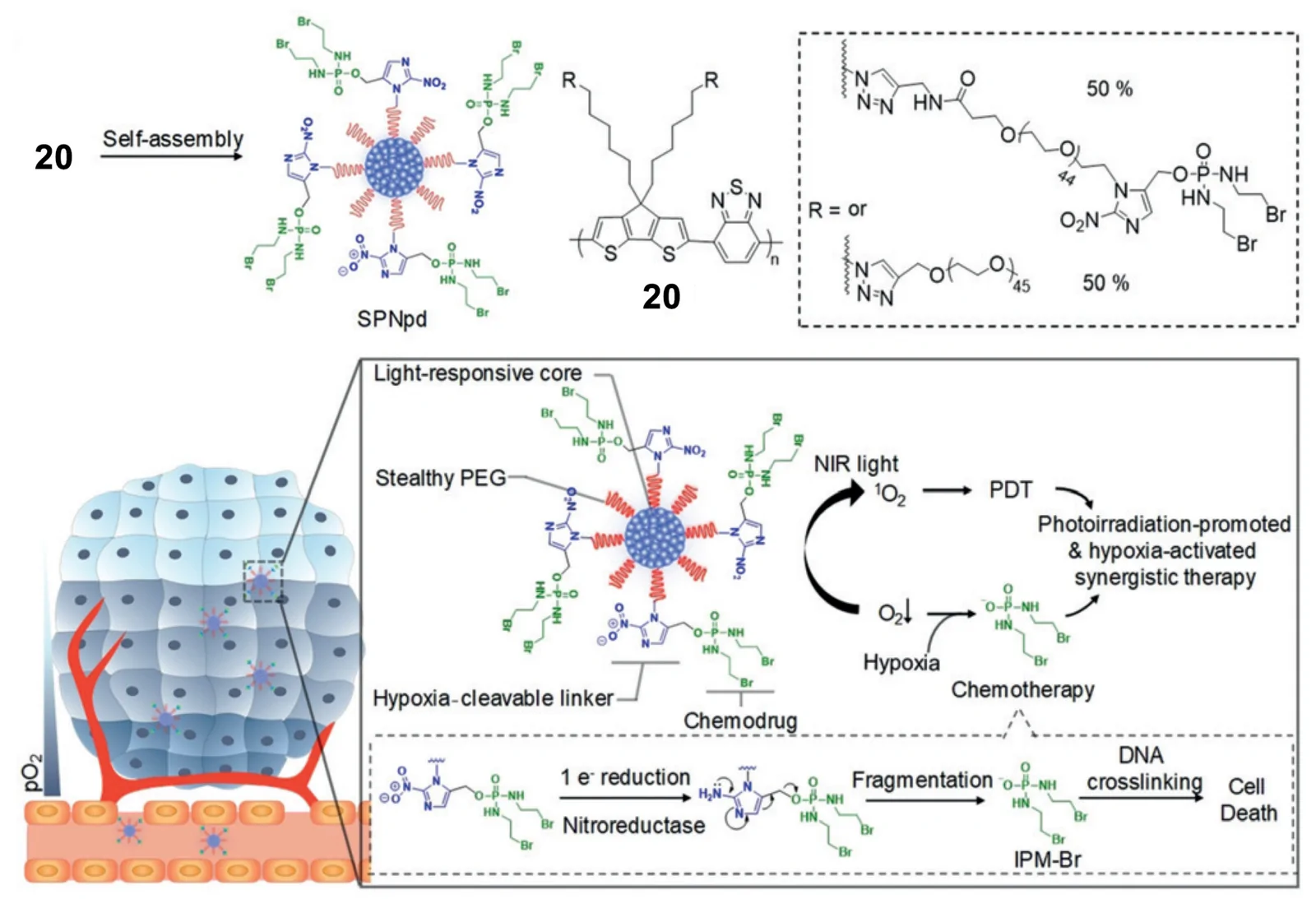
Schematic of **SPNpd** for hypoxia-activated synergistic PDT and chemotherapy. Reproduced from ref. [24]. Copyright (2019), with permission from Wiley-VCH.

**Table 1:** Comparative summary of representative hypoxia-responsive fluorescent prodrug systems.

Figure [Ref]	System	Trigger Group	Activating Enzyme/Reductive Route	O_2_ Level or Hypoxic Condition Used for Validation	Therapeutic Payload	Fluorophore	Fluorescence-Response Mode	Release/Activation Reporting	Biological Model	Extent of In Vivo Validation
Figure 12 [65]	**TPMO-Az@G/R@CD** (TPE-embedded periodic mesoporous organosilica, β-CD-capped)	Azobenzene linker **8**	Azoreductase	Hypoxic vs. normoxic cell (numerical %O_2_ not specified)	Glycine (model cargo,)	TPE (donor, green)/rhodamine B (acceptor, red)	Ratiometric FRET: TPE↑ and RhB↓; RhB/TPE ratio decreases progressively	Indirect—azo cleavage simultaneously uncaps the pores and disrupts FRET, so the ratio indirectly reports the extent of cargo release	SH-SY5Y cells	**None**—cellular level only
Figure 14 [69]	Small-molecule NIR multifunctional probe **9**	Azo bond	Azoreductase; rat liver microsomes	HeLa cells cultured under 1%, 10% and 21% O_2_; hypoxic tumour microenvironment	Melphalan analogue	NIR fluorophore	Turn-on via ICT activation	Direct—fluorophore and drug are simultaneously liberated after azoreductive cleavage	HeLa cells; tumour-bearing mice	In vivo anticancer therapy
Figure 16 [72]	Supramolecular complex **TPA-H⊂SAC4A**	Azo bonds of sulfonated azocalix[4]arene **10**	O_2_-dependent bioreduction	HeLa cells cultured under 0.1% and 20% O_2_; hypoxic tumour microenvironment	AIE-active cationic photosensitizer TPA-H	TPA-H (AIEgen; λ_ex_ 561 nm, λ_em_ 600–700 nm)	Turn-on (disruption of the electrostatic binding between TPA-H and **10**)	Direct—the therapeutic agent self-reports its own activation	HeLa cells; HeLa tumour-bearing BALB/c nude mice	In vivo fluorescence imaging and anticancer therapy
Figure 17 [74]	Acidity and hypoxia sequence-activated prodrug **12**	Dialkylamine-substituted cyanine (**11)**	Non-enzymatic hypoxia-favoured photolysis (radical pathway) under NIR light (>650 nm)	Hypoxic tumour microenvironment (numerical %O_2_ not specified)	Camptothecin	Cyanine	Wavelength shift / dual-channel readout (λ_em_ 810 → λ_em_ 530 nm	Direct – uncaging process generates the 530 nm channel concomitantly with CPT release	A549 xenograft tumour-bearing mice	In vivo dual-channel NIR fluorescence imaging of drug release
Figure 18 [78]	Theranostic small-molecule prodrug **13**	Nitrobenzene	NTR	HeLa cells cultured under 1% O_2_ (hypoxia) and normoxic conditions	SN-38	Rhodol	Turn-on	Direct—synchronized SN-38 release and rhodol release from one NTR-triggered cleavage event	HeLa cells	**None**—cellular level only
Figure 19 [79]	NTR-activatable self-immobilizing photosensitizer **14** encapsulated into mPEG-b-PDPA micelles	4-Nitrobenzyl group	NTR	4T1 cells cultured under <1%, 10% and 21% O_2_; 3D multicellular spheroid; hypoxic tumour microenvironment	Hemicyanine photosensitizer	Hemicyanine photosensitizer	Turn-on	Direct—the therapeutic agent self-reports its own activation	4T1 cells; 3D multicellular spheroid; 4T1 tumour-bearing mice	In vivo fluorescence imaging and anticancer therapy
Figure 20 [80]	Azoreductase-responsive colon-targeted prodrug **15**	Azo bond	Azoreductase from gut microbiota (rat caecal contents in vitro)	Colonic reductive environment (numerical %O_2_ not specified)	AdP (podophyllotoxin derivative)	Cyanine	Turn-on	Direct—synchronized AdP release and rhodol release after azoreductive cleavage	Rat caecal contents (in vitro); mice (oral gavage)	In vivo fluorescence imaging
Figure 21 [81]	Noncovalent theranostic prodrug **NMAC4A-CyNH_2_**	Azo bonds of nitrogen-mustard azocalix[4]arene **16**	O_2_-dependent bioreduction	HepG2 cells cultured under 0.2% and 21% O_2_; hypoxic tumour microenvironment	N,N-bis(2-chloroethyl)-1,4-benzenediamine (NM)	CyNH_2_	Turn-on (elimination of quenching effect by azo bond)	Direct—azo cleavage liberates NM and abolishes CyNH_2_ quenching	HepG2 cell; HepG2 cell xenograft tumor-bearing mice	In vivo fluorescence imaging
Figure 22 [82]	SN-38-derived azo theranostic prodrug **17**	Aryl-azo benzyl alcohol moiety	O_2_-dependent bioreduction; rat liver microsomes	Antioxidant-treated HeLa cells (numerical %O_2_ not specified); hypoxic tumour microenvironment	SN-38	SN-38	Turn-on	Direct—the drug itself is the reporter	HeLa cells; C. elegans; MDA-MB-231 cell inoculated xenograft mice; 4T1 tumour xenograft mice	In vivo fluorescence imaging and anticancer therapy
Figure 23 [83]	N-oxide-based molecular probe **18** and GYY4137 morpholine salt co-encapsulated into bis-5HT-modified liposomes (**5SGNPs**)	N-oxide group	Hypoxia-associated reduction	Normoxic vs. hypoxic HUVEC (numerical %O_2_ not specified); hypoxic pulmonary embolism microenvironment	H_2_S donor GYY4137; photosensitizer SN	SN	Turn-on	Indirect—fluorescence reports N-oxide reduction for SN release but not H_2_S release	HUVECs; pulmonary embolism mouse model	In vivo NIR-II visualization and synergistic treatment of pulmonary embolism
Figure 24 [84]	Macrophage-membrane-camouflaged M1-MPNPs encapsulating PTX prodrug **19** and AIEgen MTPETT	4-Nitrobenzyl carbonate (from 4-nitrobenzyl chloroformate)	NTR; Hypoxia-associated reduction	Treatment-induced/amplified hypoxia upon light irradiation (numerical %O_2_ not specified)	Paclitaxe; photosensitizer MTPETT	MTPETT	Constantly emissive	Indirect—the fluorescence signal can not report drug release/activation	4T1 cells; 4T1 tumour-bearing mice	In vivo fluorescence imaging and anticancer therapy
Figure 25 [24]	Semiconducting polymer nano-prodrug **SPNpd** (self-assembly of **20**)	NTR-cleavable (2-nitro-1H-imidazol-5-yl) methanol group	NTR; hypoxia exacerbated by PDT-driven O_2_ consumption under 808 nm	Treatment-amplified tumour hypoxia under 808 nm irradiation (numerical %O_2_ not specified)	IPM-Br; photosensitizer semiconducting polymer	Semiconducting polymer	Constantly emissive	Indirect—the fluorescence signal can not report drug release/activation	4T1 cells; 4T1 tumour-bearing mice	In vivo fluorescence imaging and anticancer therapy

## Data Availability

No new data were created or analyzed in this study. Data sharing is not applicable to this article.
